# The influence of Chinese scholars on global research

**DOI:** 10.1038/s41598-022-23024-z

**Published:** 2022-11-01

**Authors:** Wen-Chiao Lin, Chih-Wei Chang

**Affiliations:** Center for Condensed Matter Sciences, National Taiwan University, Taipei, 10617 Taiwan

**Keywords:** Materials science, Nanoscience and technology, Physics

## Abstract

The rise of China as a scientific research superpower has been frequently discussed in media and literature. However, past analyses are usually based on the geographical database and they ignore how the millions of emigrated Chinese students, who are now being considered the major research workforce in many countries, affect their academic outputs. Here we quantitatively analyze the contribution of Chinese scholars in physical science around the globe by their publications in a country’s papers from 2010 to 2021 as well as their citations. Contrary to common perception, we find that increasing the number of Chinese scholars does not correlate with the net publication growth or decline in their host countries before the Chinese population exceeds a critical value. On the other hand, increasing Chinese authors in a paper improves its citations. The phenomena, though anomalous, are observed in many subfields of physics across the globe. Our analysis suggests that although Chinese scholars do not change the perceived publication capabilities of many countries but may have reshaped their research culture as well as workforce distributions. The results would be valuable for R&D, higher education, and immigration policymakers.

## Introduction

The rise of China as a research superpower in science and technology has been frequently discussed in media and literature^1–5^. Since 2003, China’s publication in Science Citation Index (SCI) has increased fivefold and now China publishes more papers than any other country^6^. China has the largest number of science and engineering bachelor and master students in the world and its R&D expenditures are expected to surpass the US shortly. China has also created many funding programs, aiming to build world-class universities, attract talented scholars, and target specific disciplines for transiting to a knowledge-based economy^3,4^.

The rapid rise of China has made other top research nations (US, UK, Germany, France, and Japan) gradually lose their shares, first in numbers of publications, then in citations, and now in highly cited works^7^. The alarms have awakened many governments to take action and reform their R&D policies to meet the challenge. However, what is the proper way to stimulate the scientific performance of a nation^8^? How would R&D expenditure correlate with the scientific output? How do we measure the scientific impact of a nation^9–12^? What is the link between research and economy^13–16^? All these questions have been repeatedly discussed. Among them, many have advocated that ‘open countries have stronger science’^17,18^; that is, international collaboration via exchanges of scholars would foster impactful works^19^. The claim is supported by the observation that high mobility scholars and diverse composition of members in an institution usually generate higher citations^20–22^.

On the other hand, we note that hundreds of thousands of Chinese students migrate to many countries for their advanced education every year and they have become an important research workforce in many institutes. For example, in 2019, the numbers of Chinese students entering respective countries are about 369,500 (US), 153,800 (Australia), 109,200 (UK), 96,200 (Canada), 86,400 (Japan), 36,900 (Germany), and 30,100 (France) (Supplementary Fig. S2). These students are educated and trained in China. They could bear values and skills different from the rest of the world. For example, Chinese students’ GRE score is ranked top in the world (Supplementary Table S1), but nearly 90% of Chinese students would choose to stay in the US after receiving their PhDs^19^. How do they affect the scientific output of their host countries? How do they interact with local students/scholars? The effects have never been investigated when analyzing the scientific performance of countries.

**Figure 1:**
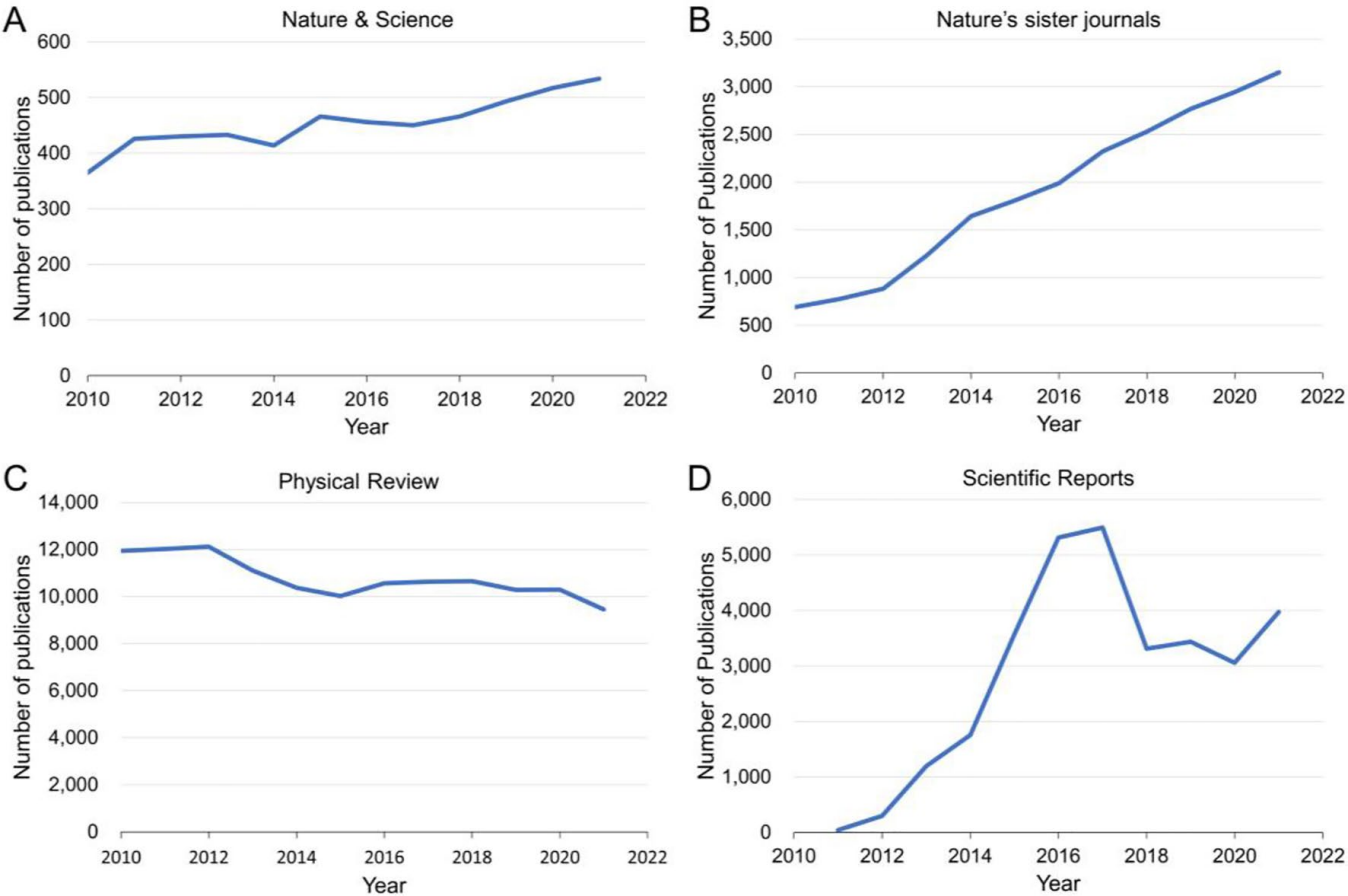
Annual publications of (**A**) Nature and Science, (**B**) Nature’s sister journals, (**C**) Physical Review family (Physical Review A, B, Letters, and X), and (**D**) Scientific Reports from 2010 to 2021. The average annual growth (*G*_0_) is 0.025, 0.128, − 0.02, and 0.108, respectively.

In this paper, we analyze the contribution of Chinese students/scholars from their publications and citations in physics journals. Because Chinese students’ strengths are in the field of physics, computer science, and engineering, choosing physics would be a representative discipline to reveal Chinese scholars’ contributions in the globe^2,12,23,24^. We attribute the first author’s affiliated country to the publication of the country. When counting publications of a country, it should be noted that many journals have variations of their publications throughout the years. For example, Fig. 1 shows the publications of two different journal families from Springer Nature from 2010 to 2021. While publications in Nature and Science have remained nearly unchanged throughout the years, Nature’s sister journals increase their publications nearly fourfold within a decade. On the other hand, families of Physical Review reduce their acceptance of publication, but large variations are seen in Scientific Reports. Adjusting the acceptance criteria is common for editors and publishers when encountering competition from other journals. For each journal, we can obtain its average annual growth (*G*_0_) from 2010 to 2021 via linear regression (Supplementary Fig. S3). We mainly choose publications in journals selected by Nature Index for our analysis. The journals selected by Nature Index usually represent quality works and they have also been used for ranking the scientific performance of countries or institutes.

Apart from high energy physics and astronomy, the first author of a paper is usually regarded as the person who contributes most to a work in the physics community. Thus quantifying Chinese scholars’ contribution to a country can be evaluated by the ratio of Chinese first authors (*r*_*1stC*_) that appears in the country’s publication; i.e., *r*_*1stC*_ = *A*_*1stC*_/(*A*_*1stC*_ + *A*_*1stNC*_), where *A*_*1stC*_ and *A*_*1stNC*_ is respectively the number of Chinese and non-Chinese first authors affiliated with a given country of a given year. We employ a computer program to identify Chinese authors by their last name. We understand that the method may also include, native-born Chinese, Taiwanese, or non-Chinese last names of identical spellings (e.g. Han). We have further checked the first name and found the overestimate is less than 1%. We have also ignored second or third authors noted as equal contributions to the first author. After counting the affiliated publications and normalizing them to the publication of 2015, the average publication growth of a country (*G*_*ave*_) can be obtained (Supplementary S2). The net publication growth (*ΔG*_*ave*_) of a nation is obtained via *ΔG*_*ave*_ = *G*_*ave*_ − *G*_0_ to make comparisons. Because the number of publications of a country is based on the affiliation of the first author, it could underestimate international collaborative works. Thus we have also analyzed the data using the whole counting method and found to reached consistent results (Supplementary Fig. S4)^25^.

## Results

### Net publication growth of each country

**Figure 2:**
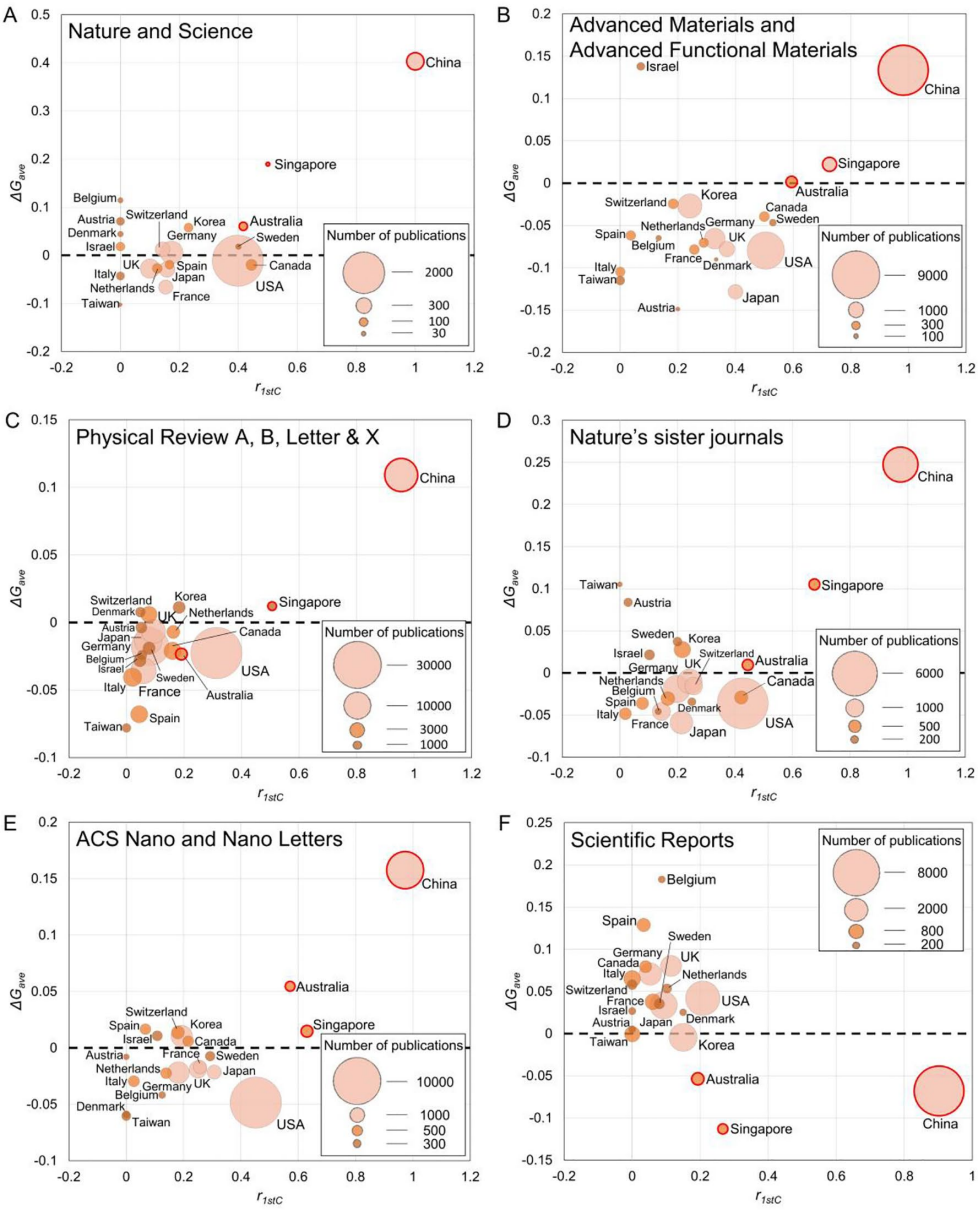
The net publication growth (*ΔG*_*ave*_) vs the ratio of Chinese first author (*r*_*1stC*_) in (**A**) Nature and Science, (**B**) Advanced Materials and Advanced Functional Materials, (**C**) Physical Review A, B, Letters, and X, (**D**) Nature’s sister journals, (**E**) ACS Nano and Nano Letters, (**F**) Scientific Reports. The area of the circle denotes the total number of publications from 2010 to 2021 of a country. China, Singapore, and Australia display notable exceptions and are denoted by the red circles.

Being the top journals in multidisciplinary sciences, Nature and Science have kept their publication nearly unchanged from 2010 to 2021 and the associated analysis would be most straightforward. Figure 2A shows *r*_*1stC*_ vs *ΔG*_*ave*_ of various countries. We notice that, except for the impressive growth of China (*ΔG*_*ave*_ > 0.4), many other countries display *ΔG*_*ave*_ ~  ± 0.1. The rapid rise of China has made the competition for publication in the top journals more intense due to the limited publication in Nature or Science. Curiously, we find that Japan and France show a decline of *ΔG*_*ave*_ ~ − 0.05 while US and Germany remain nearly unchanged. Interestingly, although Australia seems to heavily rely on Chinese scholars (*r*_*1stC*_ > 0.4) to help its growth (*ΔG*_*ave*_ = 0.06), Canada, on the other hand, does not seem to get benefited from its large portion of Chinese scholars (*r*_*1stC*_ > 0.42). Instead, we find that Korea, which has heavily invested in R&D throughout the years, displays *ΔG*_*ave*_ = 0.06. Nevertheless, except for Singapore, the correlation between *r*_*1stC*_ and *ΔG*_*ave*_ is weak.

Publishing in Nature or Science alone certainly will not reflect the improvements in research infrastructures or environments of a country. Thus we further analyze physics journals listed in Nature Index. Figure 2B shows *r*_*1stC*_ vs *ΔG*_*ave*_ published in Advanced Materials and Advanced Functional Materials of the top twenty countries. The two journals are the leading journals of material sciences and the participation of Chinese scholars varies widely across the globe. However, we find no clear correlation between *r*_*1stC*_ and *ΔG*_*ave*_. For example, Israel displays impressive growth *ΔG*_*ave*_ = 0.14 with low *r*_*1stC*_. Japan has a fairly large *r*_*1stC*_ = 0.4 but its *ΔG*_*ave*_ is the lowest. Even for *r*_*1stC*_ > 0.5 (USA, Canada, and Sweden), their *ΔG*_*ave*_ remains negative. It is until *r*_*1stC*_ > 0.6 (Australia and Singapore) that the correlation between *r*_*1stC*_ and *ΔG*_*ave*_ becomes apparent.

Compared with material sciences, the participation of Chinese scholars is much less in the traditional domain of physics. Figure 2C shows *r*_*1stC*_ vs *ΔG*_*ave*_ published in families of Physical Review (including Physical Review A, B, Letters, and X). We find that *r*_*1stC*_ < 0.2 for most countries (except for USA (*r*_*1stC*_ = 0.31) and Singapore (*r*_*1stC*_ = 0.5)). But again, no correlation between *ΔG*_*ave*_ and *r*_*1stC*_ can be identified.

Similar phenomena can be found in other journals as well. Figure 2D, E respectively shows *r*_*1stC*_ vs *ΔG*_*ave*_ in Nature’s sister journals and ACS Nano and Nano Letters. The former journals cover multidisciplinary research while the latter two generally emphasize nanoscience and nanotechnology. We notice that, except for Singapore, Australia, and Korea, many countries display *ΔG*_*ave*_ variations that are independent of *r*_*1stC*_. More similar results are also found in Applied Physics Letters, Science Advances, and Proceedings of the National Academy of Sciences (Supplementary Fig. S5).

Interestingly, an opposite phenomenon can be also observed when China’s *ΔG*_*ave*_ is negative. Since its launch in 2011 as an open-access journal, Scientific Reports has announced that it aims to assess solely the scientific validity rather than its perceived importance or novelty. Its publications may not be counted as key performance indicators in some countries. Gossips surfaced in Chinese students’ forums could further reduce their interest in submitting their manuscripts to Scientific Reports. As shown in Fig. 2F, the decline of *ΔG*_*ave*_ is most pronounced in China, Singapore, and Australia. Because of the decline of China, *ΔG*_*ave*_ > 0 is observed in many other countries. Interestingly, although traditionally the corresponding authors would decide to which journal they would submit their manuscripts, the results suggest that the Chinese authors could play some role in making the decision as well.

The results shown so far are against the common impression that Chinese students have become the major research workforce and their contributions to academic publications should have boosted *ΔG*_*ave*_ in their host countries. One may argue that the contribution of Chinese scholars could prevent the further decline of *ΔG*_*ave*_ of a country. However, we note that the *r*_*1stC*_ of Japan is either comparable to or larger than that of the UK, Germany, or France, but its *ΔG*_*ave*_ declines more. In addition, we have further explored the data by analyzing the annual publication growth (*ΔG*) vs *r*_*1stC*_ (Supplementary Fig. S6-S7). But because *ΔG* is more sensitive to short-term fluctuations of *G*_*0*_, either null or negative correlations between *r*_*1stC*_ vs *ΔG* are observed, further strengthening our finding.

**Figure 3:**
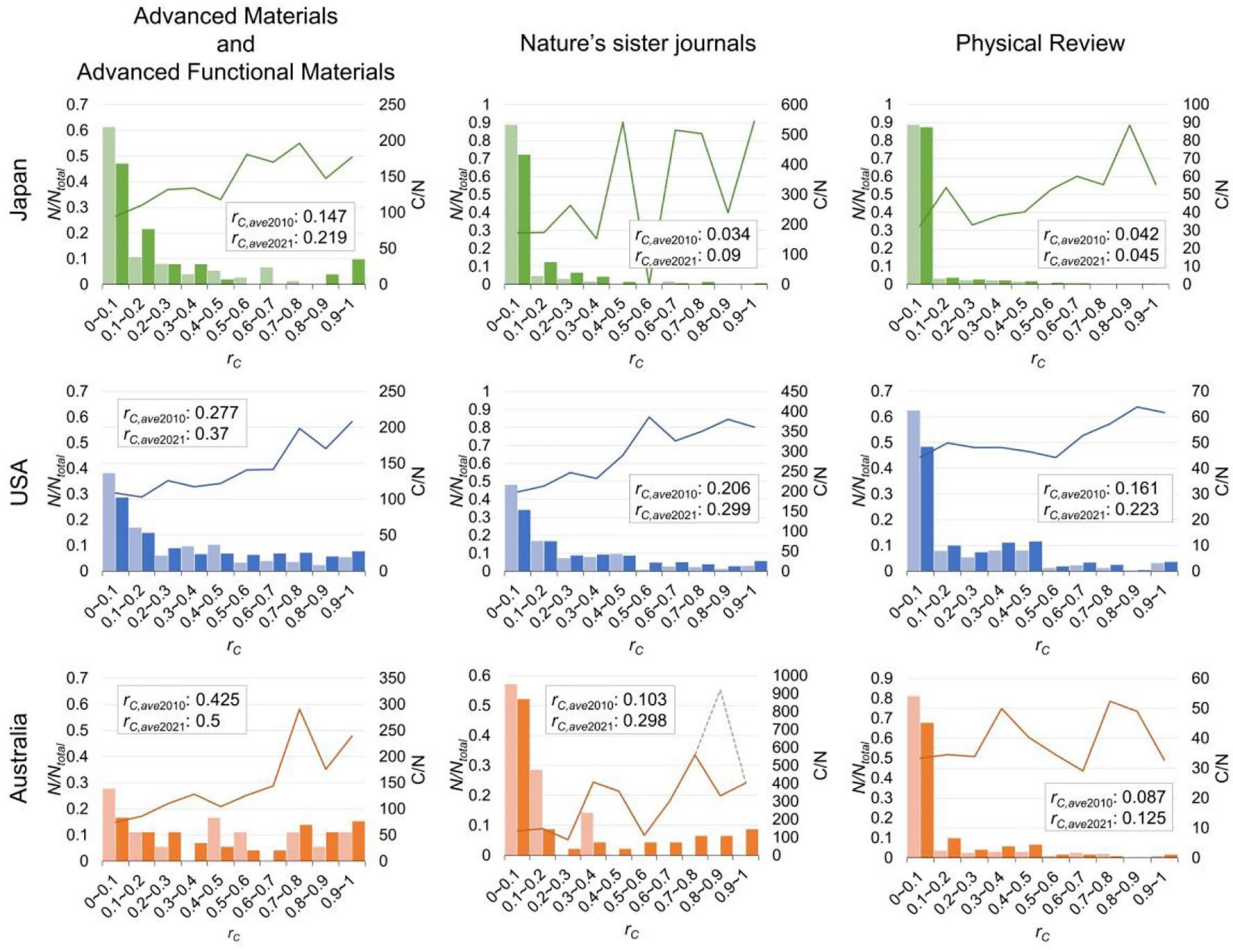
*r*_*C*_ vs *N*/*N*_total_ (columns, left y-axis) and *r*_*C*_ vs *C*/*N* (solid lines, right y-axis) in Advanced Materials and Advanced Functional Materials (left column), Nature’s sister journals (middle column), and Physical Review A, B, Letters, and X (right column), respectively contributed by Japan (top row), USA (middle row), and Australia (bottom row). The distributions of 2010 and 2021 are respectively displayed as light and dark colors. The solid lines are average citations of papers (*C*/*N*) published from 2010 to 2015. When a highly-cited paper is included^33^, the data is shown as the grey dashed line.

In contrast to many other countries’ modest or negative *ΔG*_*ave*_’s, Australia and Singapore are notable exceptions and display impressive *ΔG*_*ave*_ with *r*_*1stC*_ > 0.5 in many journals. Particularly, the two countries’ immigration and education policies have attracted a continuous inflow of Chinese students for decades, making us wonder whether they could foster their performance. To quantify the population of Chinese in a research team, we further investigate the distribution of the ratio of Chinese authors in a paper in various countries (i.e. *r*_*C*_ vs *N*/*N*_total_, where *r*_*C*_ = *A*_*C*_/(*A*_*C*_ + *A*_*NC*_), *A*_*C*_ and *A*_*NC*_ are respectively the numbers of Chinese and non-Chinese authors in a paper. For a given *r*_*C*_, *N*/*N*_total_ is the number of papers normalized by the total number of papers published in a given journal in a given year). Figure 3 shows the *r*_*C*_ vs *N*/*N*_total_ for Japan, USA, and Australia and their publications in three representative journals. Firstly, we notice that increasing Chinese contributions from 2010 to 2021 can be seen in the increase of average *r*_*C*_ (*r*_*C,*ave_) in the three journals for the three countries. Secondly, we find that Chinese scholars’ contribution is most pronounced in material sciences, as noted earlier. Thirdly, the most pronounced feature of Australia is the shift of the distribution, Chinese students have outnumbered many other students with *r*_*C,*ave_ > 0.5 in recent years.

### Chinese-dependent citation

Curiously, if the increase of Chinese students does not help Japan, USA, or UK, etc., to publish more like they do in Singapore and Australia, what would be their influence? To investigate it, we have analyzed *r*_*C*_ vs the average citations of papers (*C*/*N*) published from 2010 to 2015 (where *C* is the total citations of a given interval *r*_*C*_, accumulated from the published date to May 2022). Interestingly, Fig. 3 shows that *r*_*C*_ is correlated with *C*/*N*; that is, increasing the number of Chinese authors in a paper tends to receive more citations. For comparison, we obtain the slope of *r*_*C*_ vs *C*/*N* via linear regression and then normalize it to the citation of non-Chinese teams (i.e. *C*/*N* at *r*_*C*_ ≤ 0.1). For USA, the normalized slope is ~ 0.1, which means that increasing 10% of Chinese authors in a paper would increase the citation number by about 1% (Supplementary Fig. S8(A)). Curiously, we also find that the normalized slope varies from one country to another, showing that Australia and Singapore are usually the highest and USA is the lowest (Supplementary Fig. S8(A)). More interestingly, the unusual *r*_*C*_-dependent citation phenomena vary for different journals, with Nature’s sister journals being the most pronounced but almost diminished in journals of Physical Review (Supplementary Fig. S9).

## Discussion

**Figure 4:**
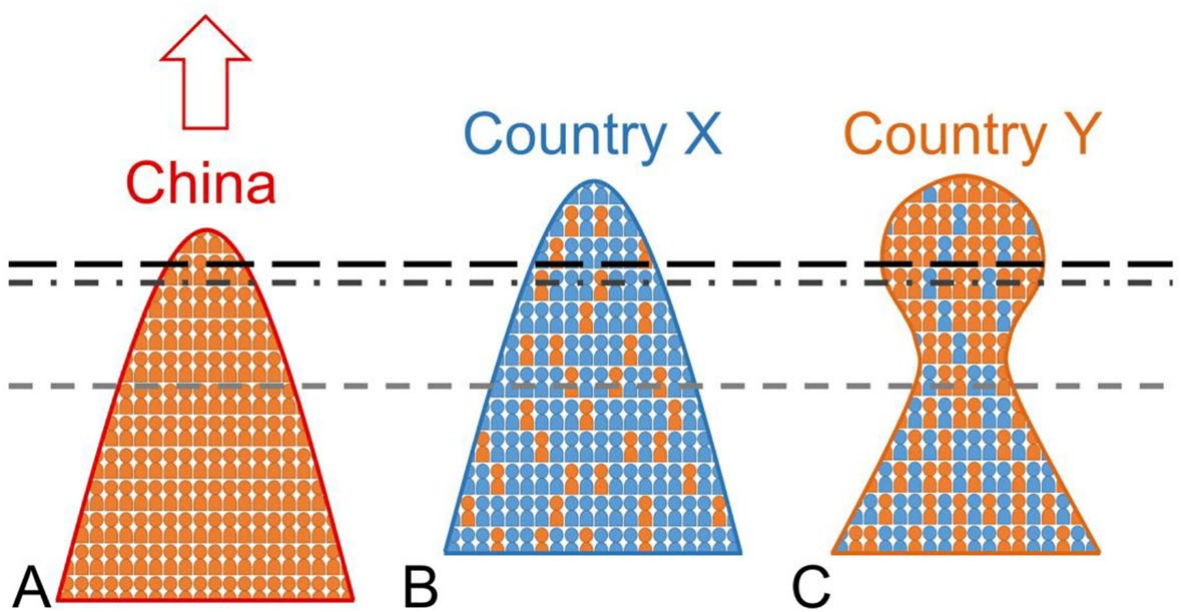
A resource distribution model of a country and the journal publication criteria for Nature and Science (black long-dashed line), Nature’s sister journals (dark grey dashed-dotted line), and other journals in Nature Index (grey dashed line). (**A**) The rise of China is denoted by the arrow. (**B**) Resource distribution of country X (e.g. USA and Germany) whose *r*_*C,*ave_ remains low and does not reshape its original distribution. Our analysis indicates that the publication criteria of Nature and Science could be lower for the two countries. (**C**) Resource distribution of country Y (e.g. Singapore or Australia) whose *r*_*C,*ave_ > 0.5 and has reshaped the distribution. Here the emigrated Chinese and domestic students are denoted by the orange and blue figures, respectively.

We provide a resource distribution model of each country, depicted in Fig. 4, to explain the observed phenomena. The resource (i.e. the combination of creativity, talents, time, funding, reputation, and luck etc.) distribution of a group of randomly-selected scientists in a country is illustrated in Fig. 4. Firstly, publishing a paper always requires large investments of resources and only a few teams are capable of putting them together at a certain time. Thus, the acceptance criteria of a journal can be represented as a bar, above which the work can be published and the scientist who contributes most will be listed as the first author of the paper. The position of the distribution may be lifted by many factors; for example, improvements in infrastructures, or the reputation of an institute or a country.

Secondly, the rise of China during the past decade is extremely rapid. As an example, China’s publications in Nature or Science were virtually negligible before 2004 and the global research was dominated by the USA, UK, Germany, France, and Japan then^26,27^. We can mathematically prove that, if the resource distribution is a convex curve, the difference of *ΔG*_*ave*_ would indicate a ranking hierarchy of journals respectively represented by the bars in Fig. 4A (Supplementary S4). Publication in Nature and Science plays an important role in creating research waves or getting immediate attention from peers. They are considered top journals among many branches of sciences. Quality works that are not accepted by the top journals may likely be published in Nature’s sister journals or other journals in Nature Index. Thus the model shown in Fig. 4A can explain why there is a difference in China’s publication growth in Nature and Science (*ΔG*_*ave*_ > 0.4), Nature’s sister journals (*ΔG*_*ave*_ = 0.25), and other journals (*ΔG*_*ave*_ < 0.15). Basically, the hierarchy of journals can be identified by their respective impact factors.

When China’s resource distribution arises, the competition for the limited space of a journal would make the *ΔG*_*ave*_ of every other country decline even if they maintain the status quo. Thus, even though Nature, Science, Nano Letters, and ACS Nano whose publications have remained nearly unchanged in the past twenty years, their acceptance bars still rise due to the competition from China.

Similar statements can be applied to journals that increase/decrease their publication after subtracting their *G*_*0*_. We can prove that the decline of *ΔG*_*ave*_ would be the same for each country if they face an equal challenge from China (Supplementary S5). The explanation, though simple, explains why many countries display *ΔG*_*ave*_ < 0 in Fig. 2A–E, as well as the *ΔG*_*ave*_ > 0 in Fig. 2F. In reality, the decline of *ΔG*_*ave*_ would not be always identical as it depends on how a country distributes its resources in detail. Thus, it can be expected that countries whose resources lie marginally above the bar or face the strongest pressure from China would decline more. From Fig. 2, we see that Taiwan, whose researchers have been constantly attracted to China, is facing the situation. On the other hand, a country may increase its *ΔG*_*ave*_ by lifting the distribution (for example, putting emphasis on education or improving research infrastructures) or reshaping it (for example, attracting more outstanding scholars). Israel and Korea have spent the highest GDP percentile on R&D in the globe and the resulting *ΔG*_*ave*_ enhancements can be observed.

We also observe an anomaly. USA and Germany display *ΔG*_*ave*_ ~ 0 in Nature and Science but *ΔG*_*ave*_ < 0 in other journals (excluding Scientific Reports). Because UK, France, and Japan do not show the similar anomaly and most papers rejected by Nature or Science should eventually be published in other journals analyzed here, we find the anomaly deserves an explanation based on the resource distribution model. Although one may say that the network of US and German scientists could help themselves in the review process of Nature and Science and increase their *ΔG*_*ave*_, it does not explain why US and Germany display *ΔG*_*ave*_ < 0 in other journals. A plausible explanation is that Nature and Science favors manuscripts from USA and Germany more, thereby lowering the acceptance criteria of the two top journals, as shown in Fig. 4B.

We now discuss the role of Chinese students. A Chinese student emigrating from China to a given country X would either replace the quota of a domestic student or add more positions to an existing distribution. But if there are no differences in the publication performance between Chinese and domestic students, the distribution will neither be lifted nor be reshaped, resulting in negligible effects on *ΔG*_*ave*_. As illustrated in Fig. 4B, it would explain why most countries display *ΔG*_*ave*_ ~  ± 0.1 variations but are independent of *r*_*1stC*_.

For Singapore and Australia, their *r*_*1stC*_ and *r*_*C,*ave_ are correlated with their high *ΔG*_*ave*_. Thus their *ΔG*_*ave*_ improvements cannot be attributed to lifting the distribution alone. Instead, a reshaped distribution shown in Fig. 4C must occur. That is, more Chinese are located in the upper part of the distribution. To confirm it, we have surveyed websites of research groups in physics departments in Singapore and found that about 10% of the lab members whose last names resemble those of Chinese are likely native Singaporeans, while the number drops to 4% when counting the *r*_*1stC*_ of Singapore. Considering Singapore’s *r*_*C,*ave_ > 0.5, the disparity could imply that, once the population of Chinese exceeds a critical value, a research group’s working style or collaboration pattern is more like a Chinese team and will not be constrained by its domestic culture. For example, Chinese PhD students may be asked by their host institute to publish a few papers before graduation. But once *r*_*C,*ave_ > 0.5, peer pressure from other Chinese could stimulate them to publish more. Notably, the phenomena are not limited to their remarkable growth in the number of publications. It has been pointed out that high citation papers are increasing more rapidly in China and Singapore than elsewhere^28^. Our analysis on the average citations of different journals of various countries also reveals a similar trend (Supplementary Fig. S8(B)).

Are the particular phenomena unique to Chinese students? We have also applied the same analysis to understand the contribution of Indian students, who have become the second-largest group of international students around the globe. However, even though the ratio of Indian students to Chinese students can be more than 1/3 in many countries, the ratio of the first authors’ publications in journals of Nature Index is much smaller, the ratio of Indian first author is approximately 1/10 to 1/5 of *r*_*1stC*_ (Supplementary Fig. S13-S15)_,_ rendering their contributions difficult to be observed. The reasons for the difference between Indian students’ and Chinese students’ publication outputs could be complex, mutually affected by their culture differences, educational backgrounds, career goals, and chances in getting academic jobs back in India or China, etc. The intriguing phenomena will be left for future studies.

So far we only discuss the resource distribution for publication in different journals. Compared with citations, neither the journal placement nor the number of publications is a good metric for measuring scientific impact, even though the initial citations can be predicted by journal placements^10^. However, it always takes many years to accumulate citations and does not provide a quick judgment of a work. Particularly, publishing in a prestigious journal is especially attractive for young scientists who are competing for grant support and getting recognized. Besides, editors who are competing for the reputation of their journals usually regard impact factors as the key metrics for their evaluation. Thus, the impact factor and the reputation of a journal are usually used as guidelines when scientists submit their manuscripts. The competition for journal placements constitutes the perceived resource distribution of publication for various countries shown in Fig. 4.

But if Chinese and non-Chinese employ identical resources for publishing in the same journal, why would Fig. 3 show a correlation between *r*_*C*_ with *C*/*N*? If citations can be regarded as a better metric for measuring scientific impact, the results of Fig. 3 suggest one possibility that the reviewers and editors of the top journals have not fully captured some essential features of good research works contributed by Chinese scholars. But if it is true, the correlation between *r*_*C*_ and *C*/*N* would be a global phenomenon without variations across countries. Neither can it explain why the *r*_*C*_-dependent citation phenomena are almost negligible in Physical Review. Another possibility is that self-citations occur more frequently within the Chinese community. Previous works have pointed out that papers in China are increasingly cited by researchers in China^29,30^. Considering a recent warning of unethical manipulation of citations in China (Supplementary S7), the self-citation could have extended to Chinese scholars outside China as well. Although it may reflect that the community of Chinese scholars exchange their information more frequently, whether the perceived citation increase generates genuine scientific impact still needs to be investigated.

When investigating whether the science of the USA is in decline in facing China’s challenge, Xie and Killewald have found that the leaky pipeline problem does not exist in STEM education but many American students could seek jobs in industries rather than staying in academics^31^. They conclude that science in the USA is not in decline but could face challenges in academic research. Our findings support their conclusion and also point out that Chinese students do not change the human resource structure in the US or those of many other countries. Furthermore, the conclusion can be further checked and replicated in the future. Due to the COVID-19 epidemic and travel policies, Chinese students’ travels or visas are more restricted in recent years. Their influence of the net publication growth can be analyzed in the future after correcting the respective recovery from COVID-19 in each country.

Even though Chinese students are talented, domestic students who choose to stay in STEM graduate school have remained highly competitive. Besides, collaboration is always needed to publish a paper in journals listed in Nature Index. Thus the influence of domestic culture must have affected Chinese students’ performance as well. Our data suggests that only when the population of Chinese students exceeds a critical value *r*_*C,*ave_ = 0.5 will they strongly boost the *ΔG*_*ave*_ of a country. The critical value of *r*_*C,*ave_ = 0.5 could suggest that Chinese students start to decouple from domestic students and carry their own research styles similar to China. It might also reveal some patterns of collaboration or culture segregation between Chinese and domestic students. Nevertheless, *r*_*C,*ave_ = 0.5 is a large number and few governments could adopt the immigration policies like those of Australia or Singapore. Nevertheless, countries that do not nurture their talents will likely jeopardize their education and economy in the future. Our results thus call for a reexamination of R&D policies from government agencies, especially when they hope to rely on Chinese students’ contributions to improve domestic research. For example, in Taiwan, there were voices of accepting more Chinese students to compensate for the reduced number of students in higher education (though the voice diminished after the increasing political tension with China). Recently, Swedish Foundation for International Cooperation in Research and Higher Education has advocated accepting more Chinese for promoting Sweden science as well as providing financial income^32^. But these policies are made without quantitative understandings of Chinese students’ potential contributions in science. Our work could thus provide valuable information for the policymakers.

## Methods

We analyze 289,899 papers from the journals (Supplementary S8) between 2010 and 2021. The titles, authors’ full names, author byline, addresses, and times cited were obtained from the Web of Science. In addition, we focused on the document type “Article” and “Letter” listed in the Web of Science and excluded group authors. The publications in multidisciplinary journals were classified using an artificial intelligence text classification program based on their titles and had 95% accuracy in selecting the physics papers.

We wrote a computer program to analyze the data. To calculate the number of Chinese authors, we first identified the first author based on the sequence of author byline and ignored equal contributions. Then the program found the first author’s affiliated country and added to the number of publications of the country. Finally, the authors’ surnames were compared with the database of 217 Chinese last names to identify likely Chinese authors.

## Supplementary Information

Supplementary Information.

## Data Availability

All data generated or analyzed during this study are included in this published article and its supplementary information files. The raw data are available from the corresponding author on reasonable request.
