# Supplementary material for: The influence of Chinese scholars on global research
Source: Sci Rep. 2022 Nov 1;12:18410. doi: 10.1038/s41598-022-23024-z (PMC9626488; doi:10.1038/s41598-022-23024-z)
Supplement: Supplementary file 1 — Supplementary Information. [file 41598_2022_23024_MOESM1_ESM.pdf]

Supplementary Information for
The Influence of Chinese Scholars on Global Research

Wen-Chiao Lin[1] and Chih-Wei Chang[1,*]
[1] Center for Condensed Matter Sciences, National Taiwan University, Taipei 10617, Taiwan

*Email: cwchang137@ntu.edu.tw

S1. Method

The data of titles, authors' full names, author byline, addresses, and times cited were obtained from the Web of Science (WoS), then classified the papers into physical science and non-physical science using MonkeyLearn (https://monkeylearn.com/), a no-code artificial intelligence text analytics. The accuracy of this classification program was evaluated through a manual check on randomly selected 200 papers and found to be better than 95%.

The following processes were conducted by our LabVIEW program. We used regular expressions to identify the country and authors. First, the program identified the country from the first author's affiliation and attributes it to the country's papers. Next, it compared the authors' last name with the Chinese last name list (the database (http://blog.udn.com/biganlinshi/2447646) has 373 Chinese surnames, which, after translating them into English, become 217 distinct surnames) and counted the number of papers with Chinese first author as well as the number of Chinese coauthors. From the data format of WoS, the authors were separated by semicolons and thus the number of authors could be obtained. The numbers of citations of the papers were collected before 2022/05/30.

The top 5 Chinese last names (Wang, Li, Zhang, Liu, Chen) constitute 30.8% of Chinese population in 2021 (https://zhuanlan.zhihu.com/p/499648090), and the top 100 common surnames already make up 85% of China's population in 2018[1]. From the population distribution of Chinese last names, we estimate that less than 1% of Chinese may be omitted in our analysis. In fact, we have manually checked 602 papers and found that 99.6% of Chinese authors are correctly identified by our computer program.

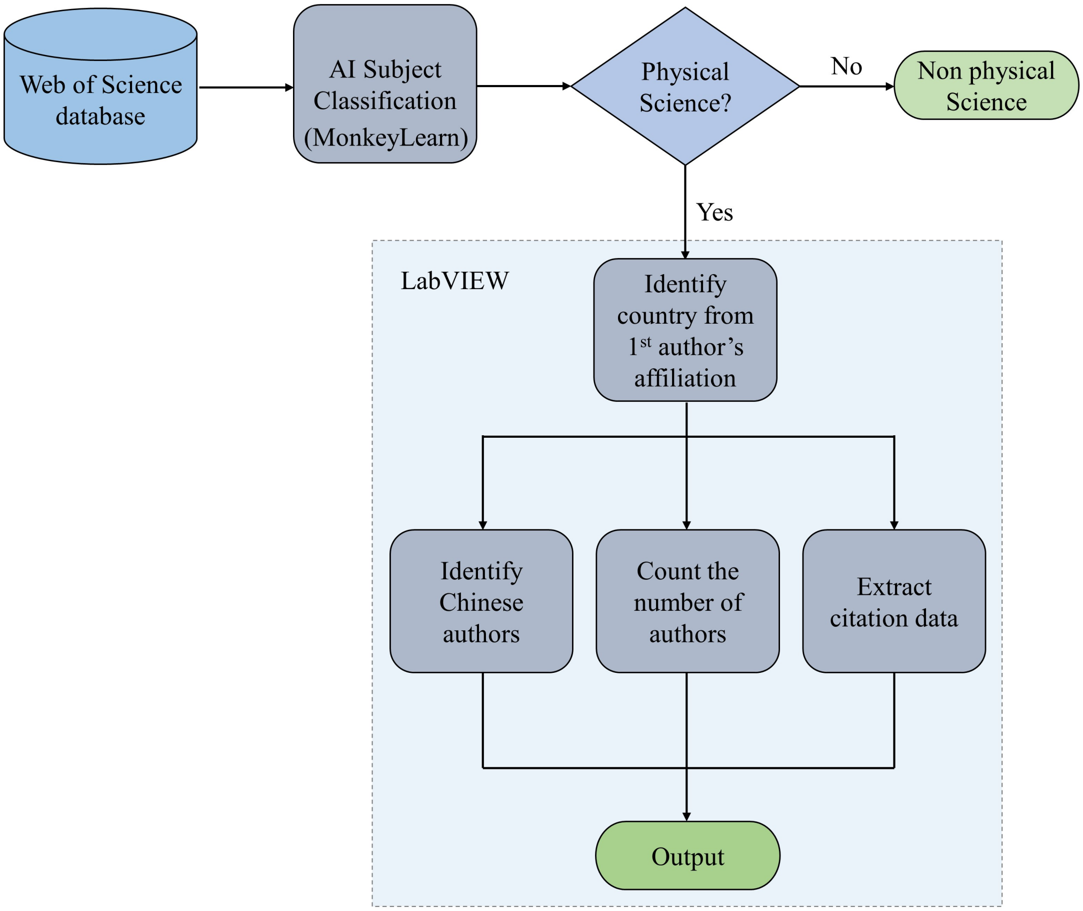

Fig. S1. Flow chart of our analysis method.

S2. Definitions of publication growth. For a given journal, we plot the number of publications (P_{year}) of a given year from 2010 to 2021 and then fit the data via linear regression. The slope of the fit ($\Delta P_{year}/\Delta t$) is then divided by the number of publications in 2015 (P_{2015}) to obtain the average publication growth (G_{ave})

$$G_{ave} = \frac{\Delta P_{year}/\Delta t}{P_{2015}} \tag{S1}$$

The net publication growth (ΔG_{ave}) of a country is obtained using

$$\Delta G_{ave} = G_{ave} - G_0 \tag{S2}$$

where G_0 is the average publication growth of a journal from 2010 to 2021.

Similarly, the annual publication growth (G_{year}) in a given year is obtained by

$$G_{year} = \frac{P_{year} - P_{year-1}}{\left(P_{year} + P_{year-1}\right)/2} \tag{S3}$$

The net annual growth is obtained using

$$\Delta G_{year} = G_{year} - \Delta G_{0,year} \tag{S4}$$

where $\Delta G_{0,year}$ is publication growth of a journal in a given year.

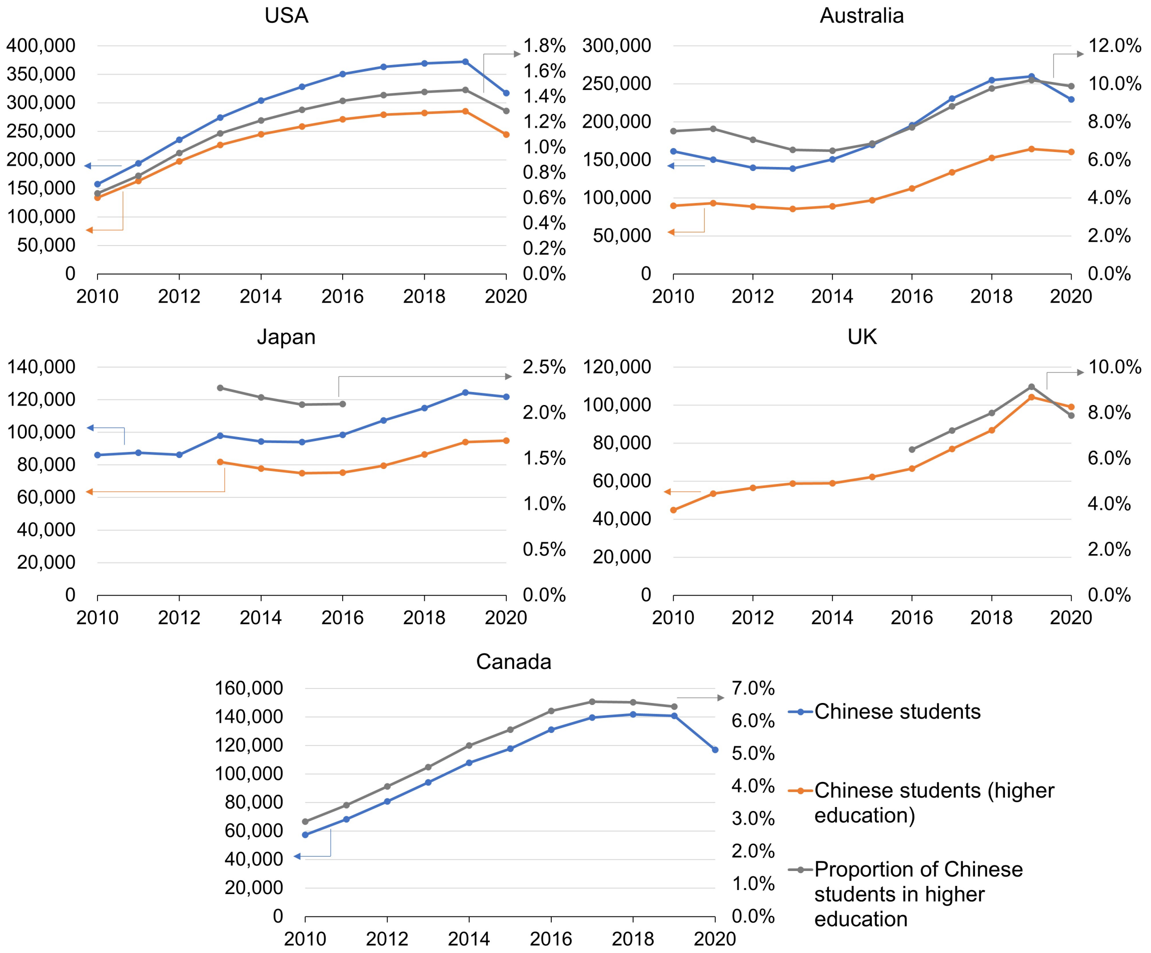

Fig. S2. Numbers of Chinese students (blue lines), Chinese students in higher education (orange lines) and proportion of Chinese students in higher education (grey lines) in the USA[2,3], Australia[4,5], Japan[6,7], UK[8], and Canada[9,10] from 2010 to 2020. Higher education institutions in Japan include graduate schools, universities, junior colleges, colleges of technology, professional training colleges, and university preparatory courses. Data in Australia are collected from enrolments; those in UK are collected from first-year entrants; and those in Canada are counted from students with valid study permit. Note that because the data of Chinese students of Canada are obtained from the study permit, the proportion of Chinese students in higher education of Canada could be overestimated.

Table S1. GRE quantitative reasoning test scores by country of citizenship between July 1, 2020, and June 30, 2021[11].

Country of Citizenship	Quantitative Reasoning Mean	Quantitative Reasoning Standard Deviation
**China**	165.6	4.9
**Taiwan**	164.1	5.7
**Korea**	161.8	6.8
**Singapore**	161.8	6.8
**Japan**	161.3	7.0
**Australia**	159.5	8.0
**Germany**	158.5	7.4
**France**	158.1	8.0
**Switzerland**	158.1	7.7
**Austria**	157.7	7.6
**Italy**	157.6	7.9
**Belgium**	157.4	7.5
**UK**	156.9	8.2
**Spain**	156.7	8.2
**Israel**	156.1	9.6
**Canada**	155.5	9.2
**Netherlands**	155.5	8.5
**Denmark**	155.3	8.1
**Sweden**	154.8	8.9
**USA**	150.7	8.2

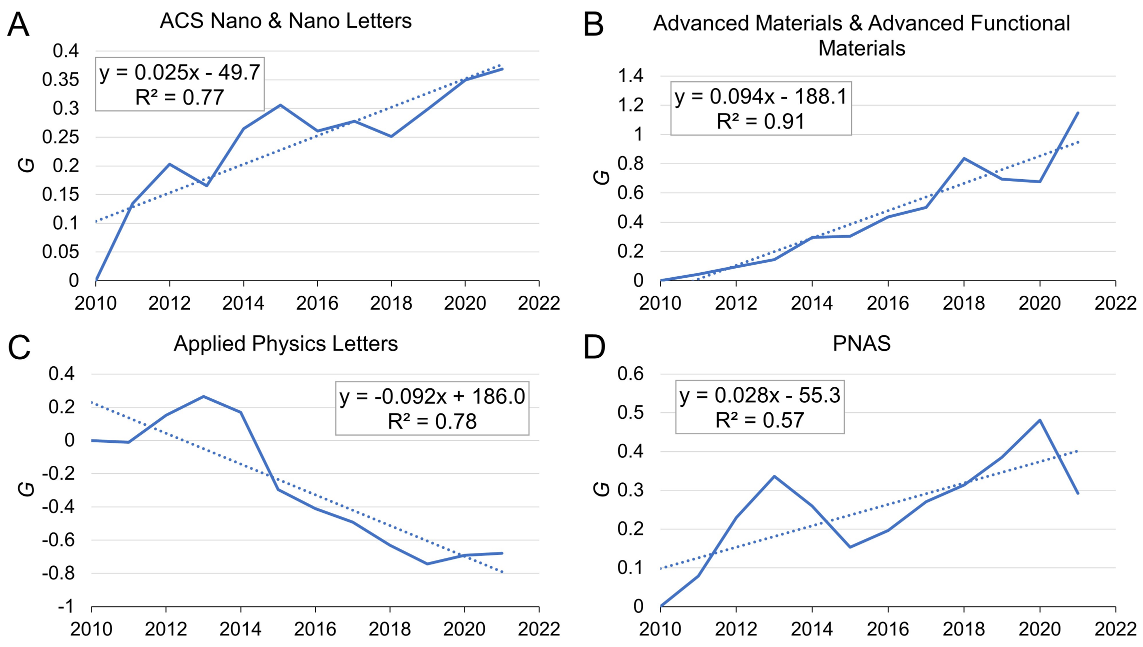

Fig. S3. The publication growth (*G*) of various journals. (A) ACS Nano and Nano Letters, (B) Advanced Materials and Advanced Functional Materials, (C) Applied Physics Letters, and (D) PNAS. The average annual growth (G_0), obtained via linear regression, is shown in each figure.

S3. The whole counting method. Apart from attributing the first authors' affiliated country as the country's publication described in the main text, we have also employed a whole counting method to calculate the publication of a country[12,13]. In a paper contributed by authors from many countries, the whole counting method would equally add one to each collaborating country. For the whole counting method, the average publication growth can be obtained from Eq. (S1). The net total publication growth ($\Delta G_{ave,whole}$) of a country is obtained in a similar way

$$\Delta G_{ave,whole} = G_{ave,whole} - G_0 \qquad \text{(S5)}$$

where the subscript denotes the whole counting method. G_0 is averaged annual growth of a journal.

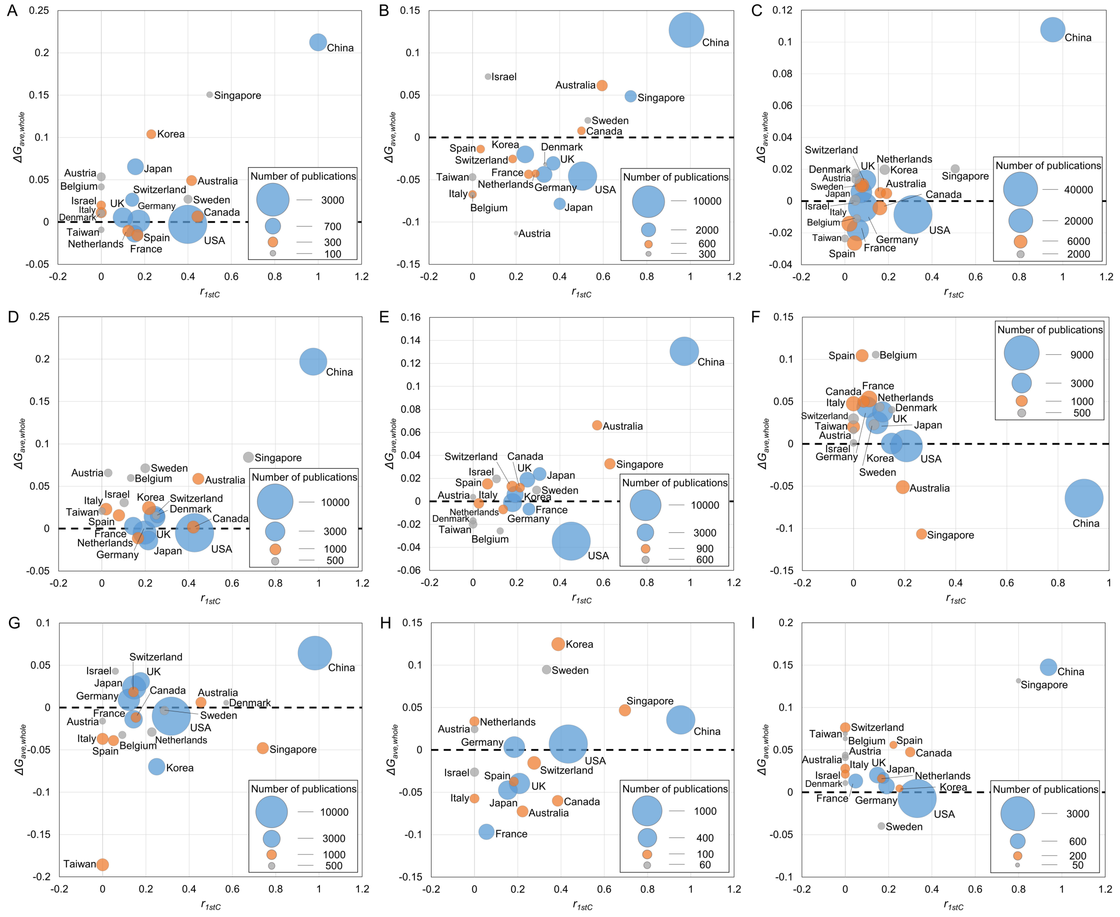

Fig. S4. r_{1stC} vs net total publication growth ($\Delta G_{ave,whole}$), obtained using the whole counting method, in (A) Nature and Science, (B) Advanced Materials and Advanced Functional Materials, (C) Physical Review, (D) Nature's sister journals, (E) ACS Nano and Nano Letters, (F) Scientific Reports, (G) Applied Physics Letters, (H) Science Advances, and (I) PNAS.

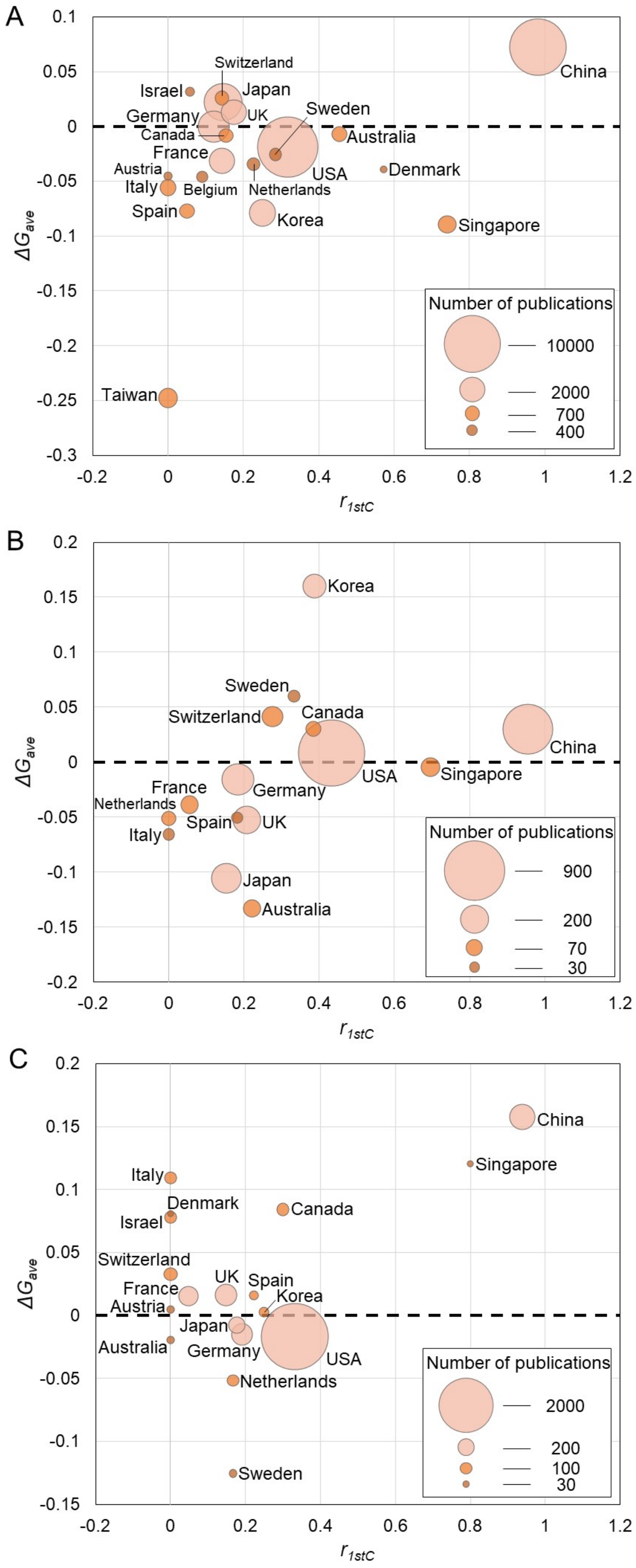

Fig. S5. r_{1stC} vs ΔG_{ave} in (A) Applied Physics Letters, (B) Science Advances, and (C) PNAS.

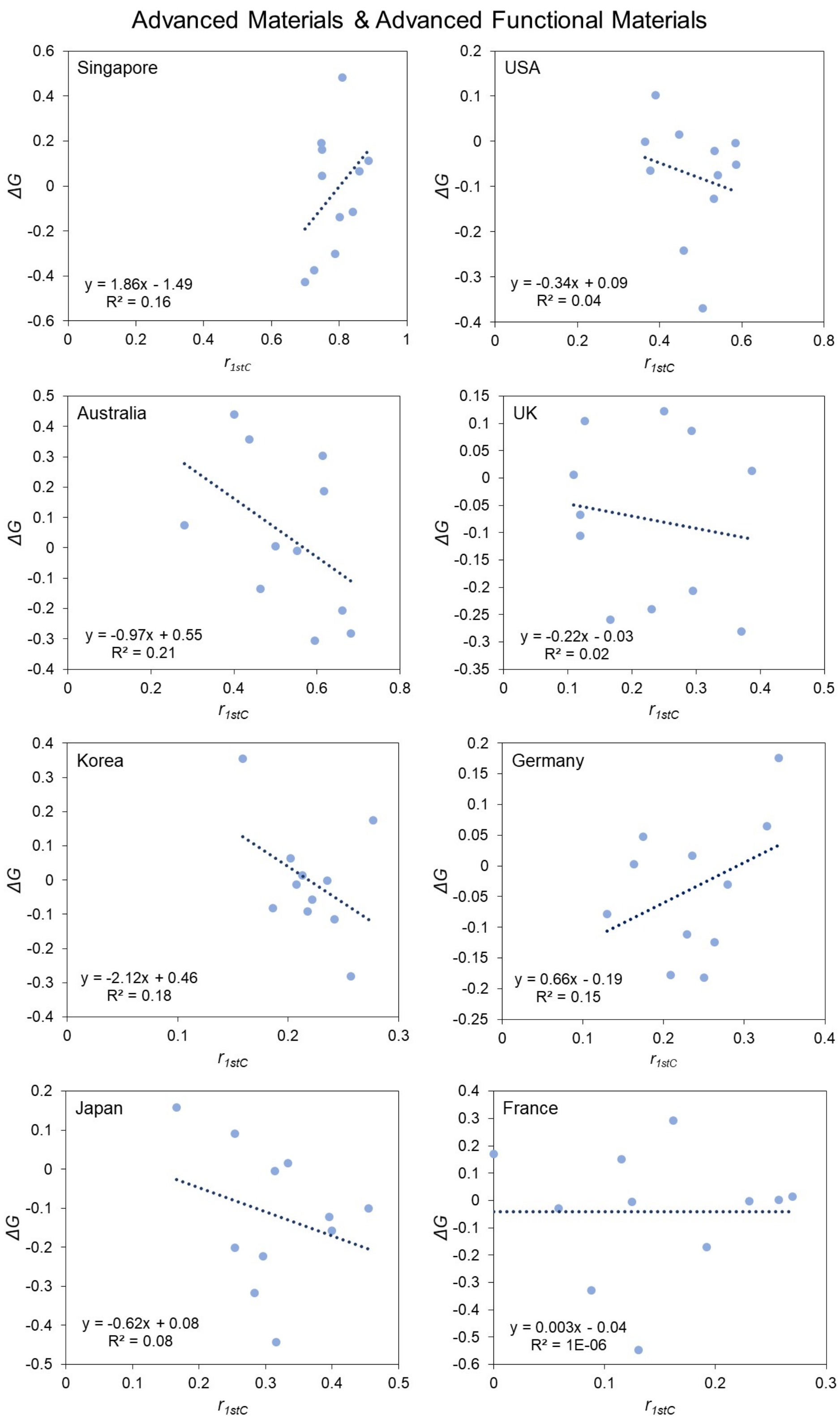

Fig. S6. r_{1stC} vs ΔG of various countries' publications in Advanced Materials and Advanced Functional Materials. The dashed lines are fitted by linear regression.

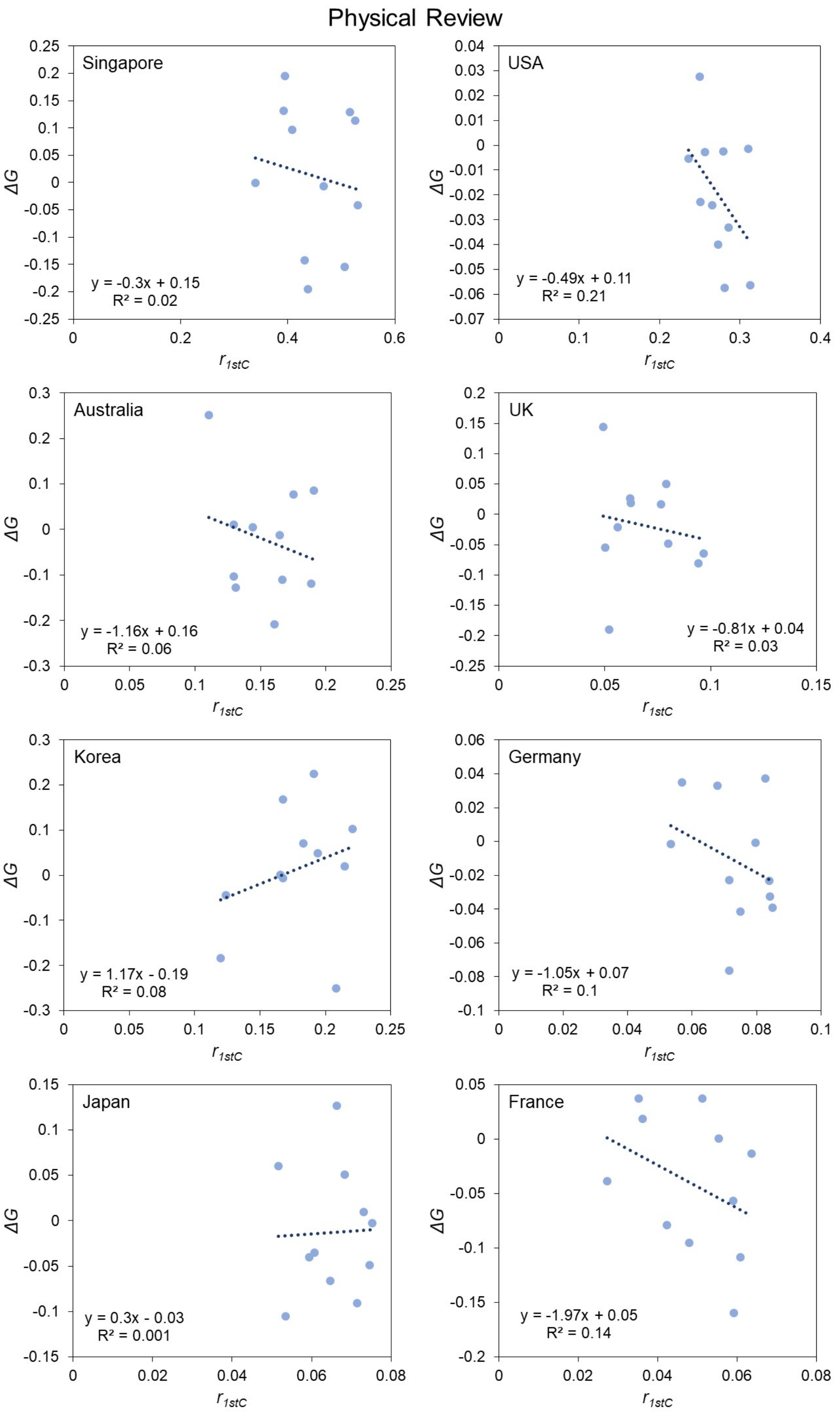

Fig. S7. r_{1stC} vs ΔG of various countries' publications in Physical Review A, B, Letters, and X. The dashed lines are fitted by linear regression.

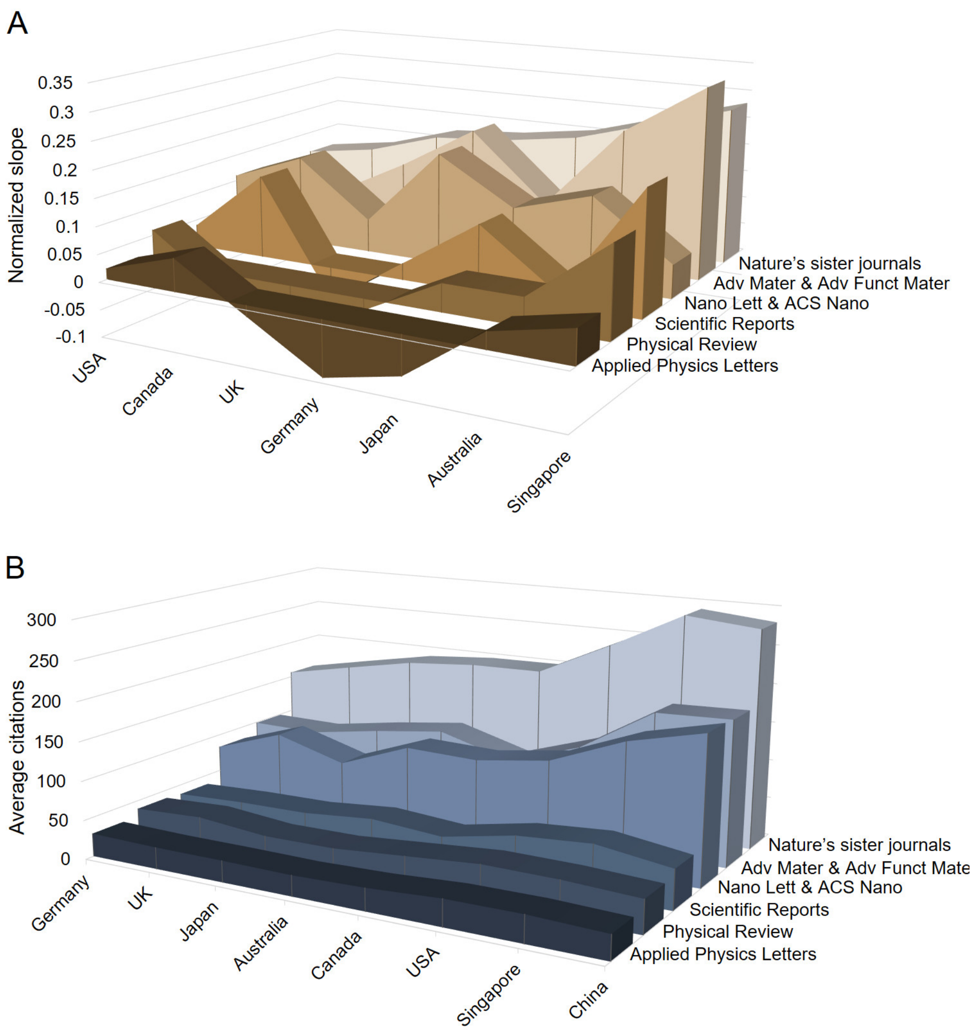

Fig. S8. (A) Normalized slope and (B) average citations of each country in various journals. When the normalized slope=0.1, it means increasing 10% of Chinese authors in a paper would increase its citation by 1%.

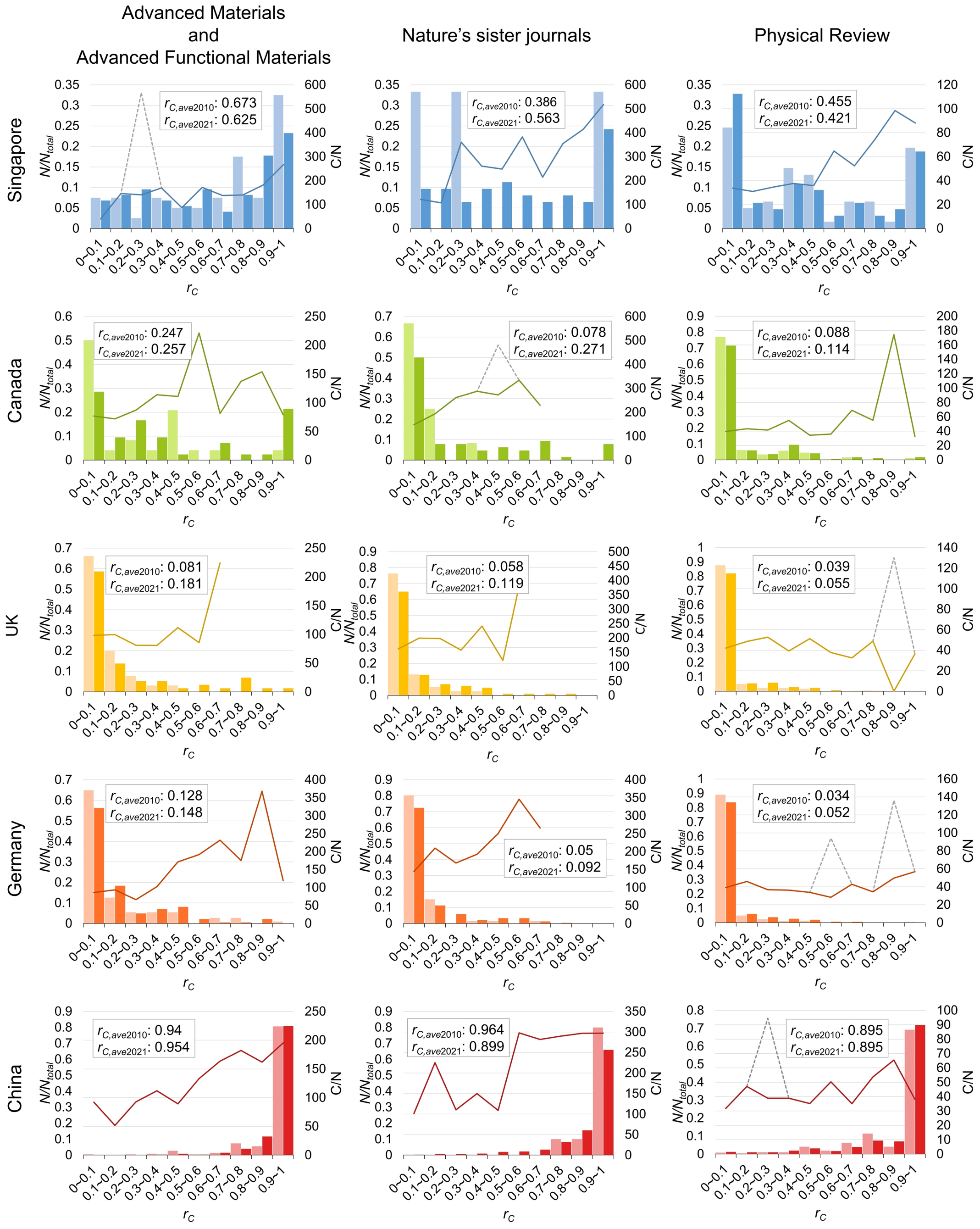

Fig. S9. r_C vs N/N_{total} (columns, left y-axis) and r_C vs C/N (solid lines, right y-axis) in Advanced Materials and Advanced Functional Materials (left column), Nature’s sister journals (middle column), and Physical Review A, B, Letters, and X (right column), respectively contributed by Singapore, Canada, UK, Germany, and China. The distributions of 2010 and 2021 are respectively displayed as light and dark colors. The solid line is average citations of papers (C/N) published from 2010 to 2015. When highly-cited papers are included[14-19], the data are shown as the grey dashed lines.

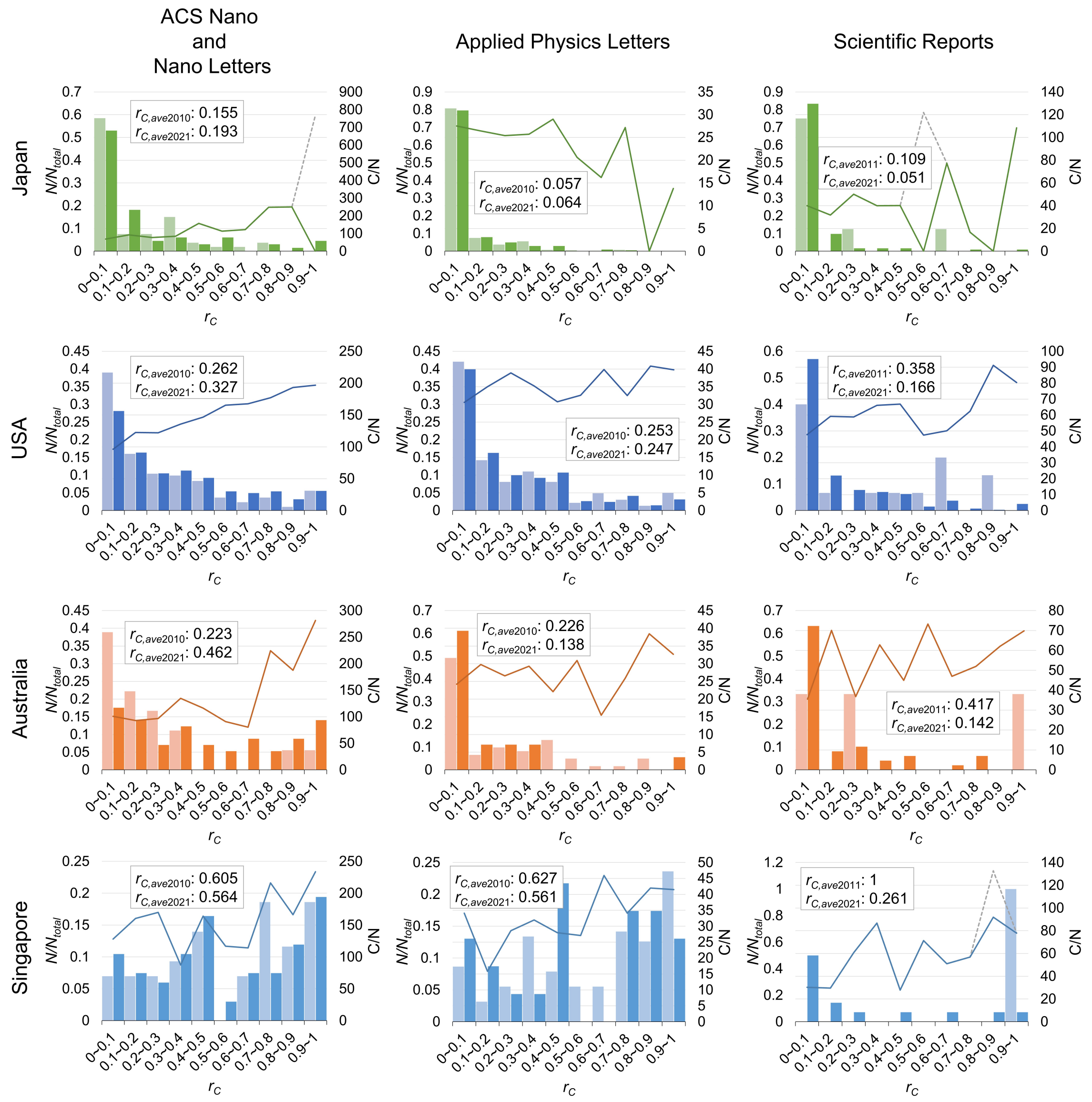

Fig. S10. r_C vs N/N_{total} in ACS Nano and Nano Letters (left column), Applied Physics Letters (middle column), and Scientific Reports (right column), respectively contributed by Japan, USA, Australia, and Singapore. The distributions of 2010 and 2021 are respectively displayed as light and dark colors. The solid line is average citations of papers (C/N) published from 2010 to 2015. When highly-cited papers are included[20-22], the data are shown as the grey dashed lines.

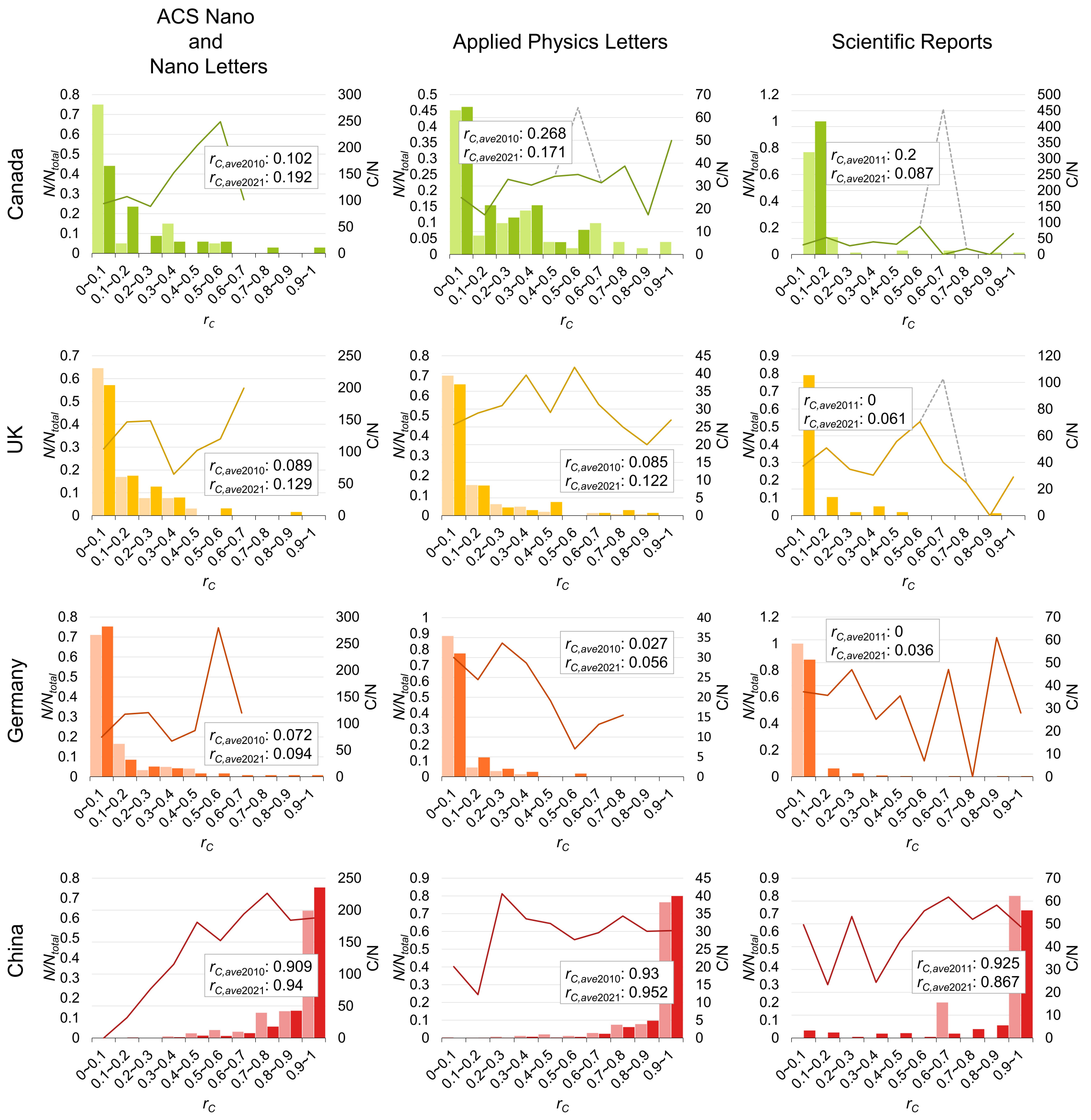

Fig. S11. r_C vs N/N_{total} (columns, left y-axis) and r_C vs C/N (solid lines, right y-axis) in ACS Nano and Nano Letters (left column), Applied Physics Letters (middle column), and Scientific Reports (right column), respectively contributed by Japan, USA, Australia, and Singapore. The distributions of 2010 and 2021 are respectively displayed as light and dark colors. The solid line is average citations of papers (C/N) published from 2010 to 2015. When highly-cited papers are included[23-25], the data are shown as the grey dashed lines.

S4. Journals' ranking hierarchy revealed from the resource distribution model. The resource distribution in China is presented by a convex function $f(y)$, as shown in Fig. S12. The rise of China lifts the function upward, resulting in a new function $f(y-a)$. If the acceptance criterion of a journal is represented as a horizontal bar in Fig. S12, the area between y_0 and y_0+c is the number of papers published in top journals such as Nature, and the area between y_1 and y_1+c' is the number of papers published in Nature's sister journals.

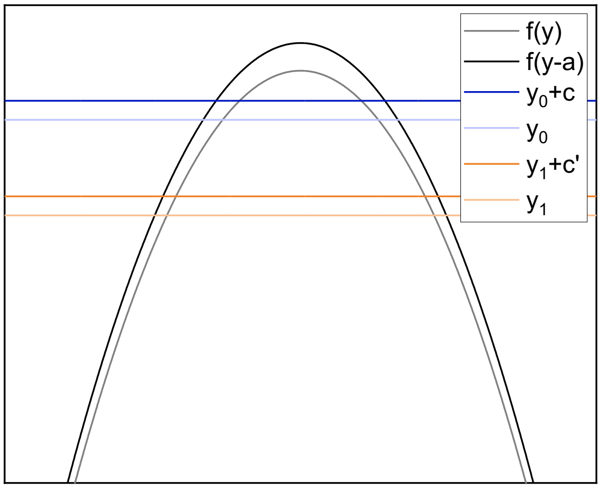

Fig. S12. Schematic illustration of resource distribution of China. The function $f(y)$ (grey) represents the distribution of the past and $f(y-a)$ (black) is the present distribution. The horizontal bars set at y_0 (light blue) and y_0+c (blue) represents the criteria for publication in Nature; y_1 (light orange) and y_1+c' (orange) are the criteria for publication in Nature's sister journals.

The net publication growth (*ΔG$_{ave}$*) of China in Nature is

$$\Delta G_{ave} = \frac{\int_{y_0}^{y_0+c}[f(y-a)-f(y)]\,dy}{\int_{y_0}^{y_0+c} f(y)\,dy} \tag{S6}$$

and that in Nature's sister journals is

$$\Delta G_{ave} = \frac{\int_{y_1}^{y_1+c'}[f(y-a)-f(y)]\,dy}{\int_{y_1}^{y_1+c'} f(y)\,dy} \tag{S7}$$

We use Taylor expansions to express $f(y-a)$:

$$f(y) - a \cdot \frac{df}{dy} + a^2 \cdot \frac{d^2 f}{dy^2} - \cdots \tag{S8}$$

Thus, the numerator of Eq. (S6) can be written as

$$\begin{aligned}\int_{y_o}^{y_0+c} [f(y-a) - f(y)]\, dy &= \int_{y_0}^{y_0+c} \left\{ \left[f(y) - a\frac{df}{dy} + a^2 \frac{d^2 f}{dy^2} \right] - f(y) \right\} dy \\ &= \int_{y_0}^{y_0+c} \left\{ \left[-a\frac{df}{dy} + a^2 \frac{d^2 f}{dy^2} \right] \right\} dy \end{aligned} \tag{S9}$$

On the other hand, the Taylor expansion of the denominator of Eq. (S6) is

$$\begin{aligned}\int_{y_0}^{y_0+c} f(y) dy &= \int_{y_0}^{y_0+c} \left[f(y_0) + \frac{df}{dy}(y - y_0) + \frac{d^2 f(y_0)}{dy^2}(y-y_0)^2 + \cdots \right] dy \\ &= f(y_0)c + \frac{df(y_0)}{dy} \int_{y_0}^{y_0+c} (y-y_0)\, dy \\ &\quad + \frac{d^2 f(y_0)}{dy^2} \int_{y_0}^{y_0+c} (y-y_0)^2 dy + \cdots \\ &= f(y_0)c + \frac{df(y_0)}{dy} \frac{1}{2} (y-y_0)^2 \Big|_{y_0}^{y_0+c} \\ &\quad + \frac{d^2 f(y_0)}{dy^2} \frac{1}{3} (y-y_0)^3 \Big|_{y_0}^{y_0+c} + \cdots \\ &= f(y_0)c + \frac{df(y_0)}{dy} \frac{1}{2} c^2 + \frac{d^2 f(y_0)}{dy^2} \frac{1}{3} c^3 + \cdots \end{aligned} \tag{S10}$$

Thus, ΔG_{ave} in Nature becomes

$$\begin{aligned}\Delta G_{ave,Nature} &= \frac{-af(y)|_{y_0}^{y_0+c} + a^2 \int_{y_0}^{y_0+c} \frac{d^2 f}{dy^2} dy}{\int_{y_0}^{y_0+c} f(y)\, dy} \\ &= \frac{-a\left(\frac{df(y_0)}{dy}\right) c + a^2 \frac{d^2 f(y_0)}{dy^2} c + \cdots}{f(y_0)c + \frac{1}{2}\frac{df(y_0)}{dy} c^2 + \cdots} \\ &= \mathrm{a} \left[- \frac{\frac{df(y_0) + a\frac{d^2 f(y_0)}{dy^2}}{dy}}{f(y_0) + \frac{1}{2}\frac{df(y_0)}{dy} c} \right] \end{aligned}$$

$$= -\mathrm{a}\frac{\frac{df(y_0 - a)}{dy}}{f\left(y_0 + \frac{c}{2}\right)} \tag{S11}$$

Similarly, ΔG_{ave} in Nature's sister journals can be obtained

$$\Delta G_{ave,NatureSister} = -a\frac{\frac{df(y_1 - a)}{dy}}{f\left(y_1 + \frac{c'}{2}\right)} \tag{S12}$$

Dividing Eq. (S11) by (S12), we obtain

$$\frac{\Delta G_{ave,Nature}}{\Delta G_{ave,NatureSister}} = \left(\frac{\frac{df(y_0 - a)}{dy}}{\frac{df(y_1 - a)}{dy}}\right) \times \left(\frac{f\left(y_1 + \frac{c'}{2}\right)}{f\left(y_0 + \frac{c}{2}\right)}\right) \tag{S13}$$

For a convex function like Fig. S8, $\frac{df(y_0 - a)}{dy}$ is larger than $\frac{df(y_1 - a)}{dy}$ and $f\left(y_1 + \frac{c'}{2}\right)$ is larger than $f\left(y_0 + \frac{c}{2}\right)$. Thus we can obtain

$$\frac{\Delta G_{ave,Nature}}{\Delta G_{ave,NatureSister}} > 1 \tag{S14}$$

which proves that ΔG_{ave} in a top journal is always larger than that of the second-tier journals. The rapid rise of China and the preference of Chinese scholars make the effect more pronounced. Thus the resource distribution model can explain why there is a difference between publications in Nature and Science (ΔG_{ave}>0.4), Nature's sister journals (ΔG_{ave}=0.25), and other journals (ΔG_{ave}<0.15) in China.

S5. The rise of China and the effect on ΔG_{ave} of other countries. To simplify the discussion, we begin by knowing that China's publications in Nature and Science was negligible in 2004, the total publications then can be written as

$$N_{total} = N_{USA} + N_{UK} + N_{Germany} + N_{Japan} + N_{France} + \dots \quad \text{(S15)}$$

or similarly,

$$\sum N_{country} = N_{total} \quad \text{(S16)}$$

Because the growth of publications in Nature and Science is nearly zero, we can obtain

$$\frac{d}{dt}\sum N_{country} = \frac{dN_{total}}{dt} = 0 \quad \text{(S17)}$$

Nowadays, China contributes a large part of publications. So we have

$$\frac{d}{dt}\left(\sum N_{country} + N_{China}\right) = 0 \quad \text{(S18)}$$

which can be rewritten as

$$\frac{d}{dt}\sum N_{country} = -\frac{dN_{China}}{dt} \quad \text{(S19)}$$

If we assume the rise of China distributes its pressure on each other country, respectively weighted by their number of publications in 2004, we then have

$$\frac{dN_{country}}{dt} = \left(\frac{N_{country,2004}}{N_{total,2004}}\right)\left(-\frac{dN_{China}}{dt}\right) \quad \text{(S20)}$$

That is

$$\left(\frac{1}{N_{country,2004}}\frac{dN_{country}}{dt}\right) = \frac{1}{N_{total,2004}}\left(-\frac{dN_{China}}{dt}\right) \quad \text{(S21)}$$

Although the above discussion is not limited to China, its most pronounced growth during the past decades clearly dominates other countries' decline. From this simple model, the decline would be equal for the major research nations prior to 2004, i.e.

$$\Delta G_{ave} = -\frac{1}{N_{total,2004}}\left(\frac{dN_{China}}{dt}\right) \tag{S22}$$

After inputting the numbers, Eq. (S22) predicted ΔGave=-0.04 for every other country. The model, though simple, still captures the essential features of Fig. 2.

S6. Influence of Indian first author. We also analyzed Indian first authors in the USA, UK, Australia, Canada, and Singapore. We use a database of 1350 Indian last names to identify Indian authors, but there could still be 20% underestimation. From Fig. S13, the numbers of Indian students in higher education in the USA, UK, and Australia are about 1/3 to 1/2 of those of Chinese students. However, the ratio of Indian first authors in Nature Index journals is approximately 1/10 to 1/5 of r_{1stC} (Fig. S14). Thus Indian students' influence on ΔG_{ave} of the above countries is much smaller.

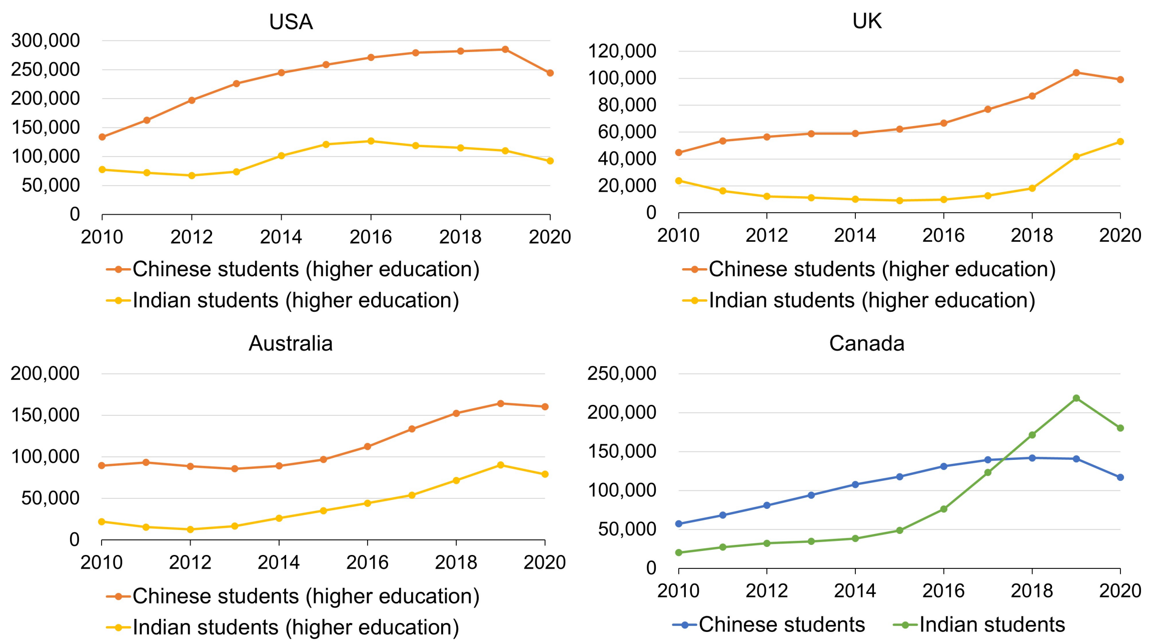

Fig. S13. Numbers of Chinese students in higher education (orange) and Indian students in higher education (yellow) in the USA, UK, and Australia, and numbers of Chinese students (blue) and Indian students (green) in Canada.

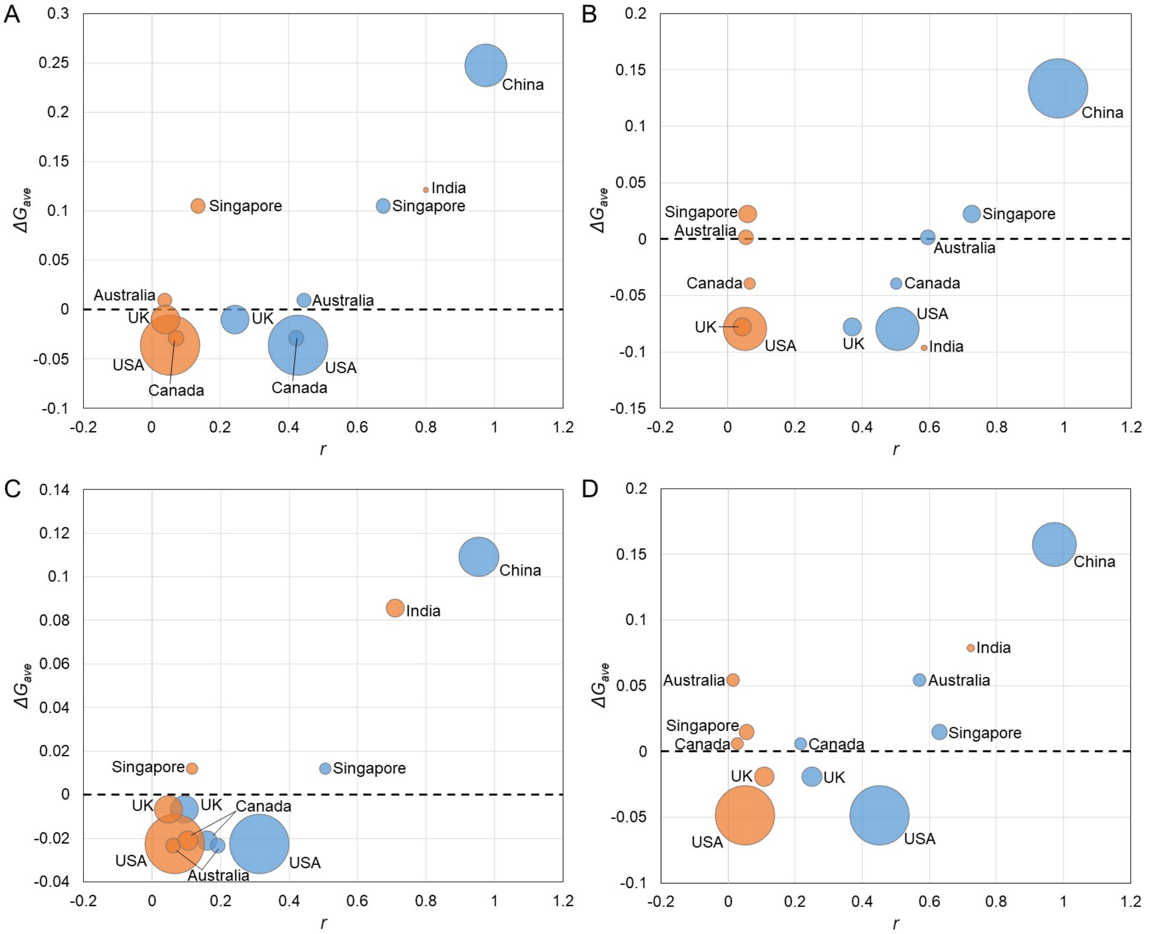

Fig. S14. The net publication growth (ΔG_{ave}) vs the ratio of Chinese first author (blue) and Indian first author (orange) in (A) Nature's sister journals, (B) Advanced Materials and Advanced Functional Materials, (C) Physical Review family (Physical Review A, B, Letters, and X), and (D) ACS Nano and Nano Letters.

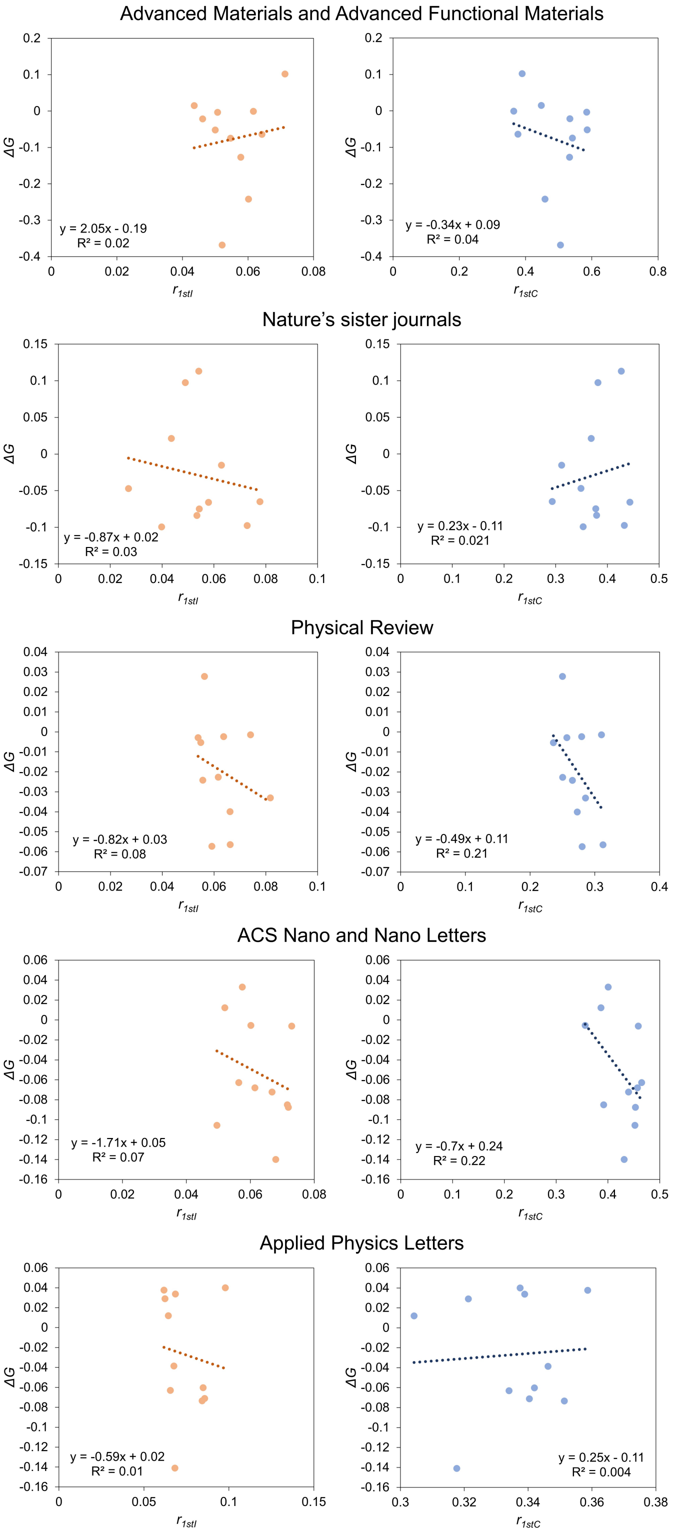

Fig. S15. Comparisons of the annual publication growth (ΔG) vs ratio of Indian first author (r_{1stI}) and r_{1stC} in the USA in Advanced Materials and Advanced Functional Materials, Nature’s sister journals, Physical Review family (Physical Review A, B, Letters, and X), ACS Nano and Nano Letters, and Applied Physics Letters.

S7. (from a website of the University of Science and Technology of China) Irregular citations are becoming a new type of scientific research dishonesty

https://kyb.ustc.edu.cn/2021/1213/c20734a538818/page.htm

S8. List of journals investigated in this work

Nature's sister journals

Nature Astronomy
Nature Catalysis
Nature Chemistry
Nature Electronics
Nature Energy
Nature Materials
Nature Nanotechnology
Nature Photonics
Nature Physics
Nature Communications

Multidisciplinary journals

Nature
Nature Communications
Science
Science Advances
Scientific Reports
Proceedings of the National Academy of Sciences of the United States of America

Nature Index journals: Physical Sciences

ACS Nano
Nano Letters
Advanced Functional Materials
Advanced Materials
Applied Physics Letters
Physical Review A
Physical Review B
Physical Review Letters
Physical Review X

References

1. Wikipedia, List of most common surnames in Asia. https://en.wikipedia.org/wiki/List_of_most_common_surnames_in_Asia. Accessed 23 August 2022.
2. Institute of International Education, International Students by Academic Level and Place of Origin, 2000/01-2020/21. https://opendoorsdata.org/data/international-students/academic-level-and-places-of-origin/ (2021). Accessed 6 April 2022.
3. Education Data Initiative, College Enrollment & Student Demographic Statitics. https://educationdata.org/college-enrollment-statistics (2022). Accessed 22 August 2022.
4. Department of Education, Skills and Employment, International Student Data. https://www.dese.gov.au/international-data/resources/international-student-data-december-2021-data-tables (2022). Accessed 6 April 2022.
5. Department of Education and the Department of Employment and Workplace Relations, Student Data. https://www.dese.gov.au/higher-education-statistics/student-data (2022). Accessed 22 August 2022.
6. Japan Student Services Organization, Result of an Annual survey of International Students in Japan. https://www.studyinjapan.go.jp/en/statistics/zaiseki/ (2022). Accessed 6 April 2022.
7. Research Institute for Higher Education, Hiroshima University, Statistics of Japanese Higher Education. https://rihe.hiroshima-u.ac.jp/en/statistics/synthesis/ (2016) . Accessed 23 August 2022.
8. Higher Education Statistics Agency, Where do HE students come from? https://www.hesa.ac.uk/data-and-analysis/students/where-from (2022). Accessed 6 April 2022.
9. Immigration, Refugees and Citizenship Canada, Study permit holders with valid permit as of December 31 by country of citizenship. https://www.cic.gc.ca/opendata-donneesouvertes/data/IRCC_M_TRStudy_0009_E.xls (2021). Accessed 6 April 2022.
10. Statistics Canada, Postsecondary enrolments, by field of study, registrarion status, program type, credential type and gender. https://www150.statcan.gc.ca/t1/tbl1/en/tv.action?pid=3710001101 (2021). Accessed 23 August 2022.
11. ETS, A Snapshot of the Individuals Who Took the GRE General Test. https://www.ets.org/gre/snapshot (2022). Accessed 6 April 2022.
12. Huang, M. H., Lin, C. S. & Chen, D. Z. Counting Methods, Country Rank Changes, and Counting Inflation in the Assessment of National Research Productivity and Impact. *J. Am. Soc. Inf. Sci. Tec.* **62**, 2427-2436 (2011).
13. Waltman, L. & van Eck, N. J. Field-normalized citation impact indicators and the choice of an appropriate counting method. *J. Informetr.* **9**, 872-894 (2015).
14. Li, H. *et al.* From Bulk to Monolayer MoS2: Evolution of Raman Scattering. *Adv. Funct. Mater.* **22**, 1385-1390 (2012).
15. Liu, D. Y. & Kelly, T. L. Perovskite solar cells with a planar heterojunction structure prepared using room-temperature solution processing techniques. *Nat. Photonics* **8**,

133-138 (2014).
16. Lawrence, M. *et al.* Manifestation of PT Symmetry Breaking in Polarization Space with Terahertz Metasurfaces. *Phys. Rev. Lett.* **113**, 093901 (2014).
17. Liu, C. X. *et al.* Oscillatory crossover from two-dimensional to three-dimensional topological insulators. *Phys. Rev. B* **81**, 041307 (2010).
18. Sun, Y., Wu, S. C., Ali, M. N., Felser, C. & Yan, B. H. Prediction of Weyl semimetal in orthorhombic MoTe2. *Phys. Rev. B* **92**, 161107 (2015).
19. Wan, X. G., Turner, A. M., Vishwanath, A. & Savrasov, S. Y. Topological semimetal and Fermi-arc surface states in the electronic structure of pyrochlore iridates. *Phys. Rev. B* **83**, 205101 (2011).
20. Chang, K. *et al.* MoS2/Graphene Cocatalyst for Efficient Photocatalytic H-2 Evolution under Visible Light Irradiation. *ACS Nano* **8**, 7078-7087 (2014).
21. Zhou, H. *et al.* Leaf-architectured 3D Hierarchical Artificial Photosynthetic System of Perovskite Titanates Towards CO2 Photoreduction Into Hydrocarbon Fuels. *Sci. Rep.* **3**, 1667 (2013).
22. Li, J. *et al.* A stable solution-processed polymer semiconductor with record high-mobility for printed transistors. *Sci. Rep.* **2**, 754 (2012).
23. Hu, A. *et al.* Low temperature sintering of Ag nanoparticles for flexible electronics packaging. *Appl. Phys. Lett.* **97**, 153117 (2010).
24. Sun, S. H. *et al.* Single-atom Catalysis Using Pt/Graphene Achieved through Atomic Layer Deposition. *Sci. Rep.* **3**, 1775 (2013).
25. Lan, R., Irvine, J. T. S. & Tao, S. W. Synthesis of ammonia directly from air and water at ambient temperature and pressure. *Sci. Rep.* **3**, 1145 (2013).
